# Computational Optimization of a Stent for the Femoropopliteal Artery

**DOI:** 10.1007/s10439-025-03968-9

**Published:** 2026-01-16

**Authors:** Alexey Kamenskiy, Jason MacTaggart, Anastasia Desyatova

**Affiliations:** 1 Department of Biomechanics, University of Nebraska Omaha, Omaha, NE, USA; 2 Department of Surgery, University of Nebraska Medical Center, Omaha, NE, USA

**Keywords:** Peripheral artery disease, Stent, Finite element analysis, Limb flexion, Optimization

## Abstract

**Purpose:**

Clinical outcomes of peripheral artery disease (PAD) stenting, particularly in the highly dynamic regions of the femoropopliteal artery at the adductor hiatus and behind the knee, leave significant room for improvement. Despite the availability of various stent designs, few are capable of accommodating the severe deformations induced by limb flexion at these locations without causing adverse stent-artery interactions.

**Methods:**

This study employed finite element analysis and response surface methodology to optimize the geometric design of nitinol PAD stents, with the objectives of improving stent-artery apposition, reducing arterial wall stress, minimizing stress concentrations, and decreasing arterial pinching under limb flexion-induced deformations. Five geometric parameters - strut width, thickness, amplitude, number, and link amplitude - were analyzed to assess their influence on stent performance.

**Results:**

Strut width, thickness, amplitude, and the number of struts significantly impacted arterial stress and apposition, while link amplitude had an insignificant effect. We identified two optimized stent configurations that achieved > 97% stent-artery apposition, < 0.6% of the artery with stress > 100 kPa, an average arterial stress of < 29 kPa, and pinching of < 1.15. The findings revealed that lower strut amplitude and reduced strut cross-sections improved apposition and stress distribution but required careful balancing to minimize arterial pinching and maintain structural integrity.

**Conclusion:**

This study underscores the potential of multi-objective optimization in stent design, paving the way for PAD stents that more effectively accommodate femoropopliteal biomechanics and promote favorable mechanical conditions for healing.

## Introduction

Peripheral artery disease (PAD) of the femoropopliteal artery (FPA) is most often characterized by atherosclerotic lesions that restrict blood flow to the lower extremities. It is associated with leg pain, non-healing ulcers, and, in severe cases, may lead to amputation [1]. Globally, over 200 million people suffer from PAD, with treatment costs in the United States alone exceeding $21 billion annually [2, 3]. This substantial expense is primarily attributed to the high rate of treatment failures [4–8]. Angioplasty with stent placement is the first-line therapy for severe PAD in most clinics. Compared to bypass grafting [9], it is minimally invasive and offers significantly shorter recovery times. During balloon angioplasty, a catheter is typically inserted through the femoral artery in the groin and guided to the site of stenosis. Once positioned, the balloon is inflated to compress the plaque against the arterial wall, enlarging the lumen and improving blood flow to distal tissues. However, acute flow-limiting arterial dissections occur in 30 to 40% of cases [10, 11], requiring bailout stenting [12] to push the dissection septum against the artery wall, preventing it from obstructing the lumen. Despite stent placement, 30 to 50% of treated arteries develop hemodynamically significant restenosis within 1 to 3 years [9, 13], necessitating repeat interventions [14–17]. The consequences of failed treatments include intractable pain, non-healing ulcers, amputation, and long-term disability.

Drug-eluting devices were initially expected to revolutionize endovascular treatment of PAD by improving short-term patency outcomes. Recent randomized trials, including EMINENT and SPORTS, demonstrated higher 12-month patency rates for polymer-based paclitaxel-eluting stents such as Eluvia (83–87%) compared with bare-metal stents (74–75%) [18, 19]. However, these benefits have not translated into improved clinical outcomes. In long-term follow-up, aneurysmal degeneration has been reported in up to 8% of patients treated with Eluvia [20, 21], raising concerns about local vessel toxicity and converting previously stable cases into those requiring closer surveillance for thrombotic and bleeding complications. This phenomenon is likely multifactorial: the abrasive mechanical environment of the femoropopliteal artery can injure the vessel wall, and when combined with prolonged local paclitaxel release - which suppresses smooth muscle cell proliferation and delays healing - the result may be localized weakening of the arterial wall and aneurysm formation. Moreover, the large-scale SWEDEPAD 1 and SWEDEPAD 2 trials - including more than 3,500 patients - showed that paclitaxel-coated devices did not reduce major amputation rates or improve disease-specific quality of life compared with uncoated devices, and were associated with higher five-year mortality [22, 23]. Together, these data suggest that although drug-eluting stents may transiently enhance angiographic patency, they do not improve long-term limb outcomes and may introduce additional safety concerns. Importantly, once the drug is eluted - typically within weeks to months after implantation [24] - the stent behaves mechanically as a bare-metal device, remaining susceptible to the same deformation-driven failures and restenosis mechanisms. Thus, rather than mitigating the effects of vessel injury, prolonged drug release in an unoptimized mechanical environment may exacerbate them - underscoring that durable success in PAD treatment requires addressing the underlying biomechanical causes of stent failure.

The high failure rates of PAD interventions are therefore likely driven by the complex biomechanical environment that subjects arteries and stents to significant deformations during limb flexion [25, 26]. These deformations include axial compression (foreshortening) [27], bending [7], twisting [28], and pinching [29], with the most severe deformations occurring at the adductor hiatus (AH) and behind the knee. Historically, the magnitude of these deformations has been measured using arterial side branches identified through conventional clinical imaging modalities such as fluoroscopy, magnetic resonance imaging, and computed tomography angiography [30–32]. These imaging studies have demonstrated relatively mild deformations, including 4–12% axial compression, a 22–72 mm radius of curvature, 2–4°/cm twist, and a pinching aspect ratio of 1.05–1.13 [33]. However, more recent perfused cadaver studies have challenged these findings by employing specially designed inter-arterial markers to quantify deformations without relying on side branches for measurements. These studies revealed that localized deformations are significantly more severe [7, 25–29], reporting 9–25% axial compression, an 8–27 mm radius of curvature, 8–26°/cm twist, and a pinching aspect ratio of 1.14–1.35.

Bench-top evaluations of commercially available stents for PAD repair subjected to such severe limb flexion-induced deformations have demonstrated the inability of most devices to accommodate these mechanical demands. These tests revealed stent’s structural instability, characterized by global buckling and severe diameter pinching [34]. A perfused cadaver study further confirmed that while some stents could withstand certain deformation modes, no device could adequately accommodate all limb flexion-induced deformations [35]. Instead, these stents exacerbated or restricted natural arterial biomechanics, not only within the stented segment but also in adjacent regions. This mismatch between the behavior of stents and the natural biomechanics of the femoropopliteal artery during limb flexion results in adverse stent-artery interactions, causing abrasive damage to the endothelial surface. Such damage correlates with the loss of primary patency [36], likely due to repeated arterial wall injury compounded by stent strut fracture [33, 37, 38], ultimately leading to restenosis and reconstruction failure [39, 40]. Furthermore, several stent designs have demonstrated poor arterial apposition, increasing the likelihood of thrombotic events and in-stent restenosis [41–44]. This underscores the urgent need for an optimized stent design capable of accommodating the severe biomechanical deformations of the femoropopliteal artery during limb movement while minimizing arterial injury.

Although multiple studies have investigated stent implantation in the FPA [37, 45–49], few have focused on the mechanical performance of PAD stents and their interactions with the arterial wall to inform an optimized stent design [41, 50, 51]. Our current study aims to determine these optimal design characteristics by conducting parametric computational studies and by subjecting stents to the combined axial compression, torsion, and bending deformations that the FPA at the AH experiences during acute limb flexion [7, 26, 28]. The primary objectives of the study were represented through four quantitative outcome measures that reflect clinically relevant mechanisms of restenosis and flow impairment: minimizing mean arterial wall stress, minimizing the arterial surface area exposed to stresses above 100 kPa (a threshold associated with tissue damage [52]), maximizing stent-artery apposition, and minimizing luminal pinching. These measures collectively capture the dual goals of reducing arterial injury while preserving blood flow. To achieve this, we utilized a multi-objective optimization approach, employing response surface methodology (RSM) to identify the optimal stent design parameters. The resulting computational models predict maximum stent-artery apposition while minimizing stress concentrations in the artery wall and preventing excessive pinching. Our findings provide a pathway toward developing a stent capable of enhancing clinical outcomes by improving mechanical compatibility and reducing failure rates of PAD interventions.

## Materials and Methods

### A Parametric Model of the FPA Stent

**Fig. 1:**
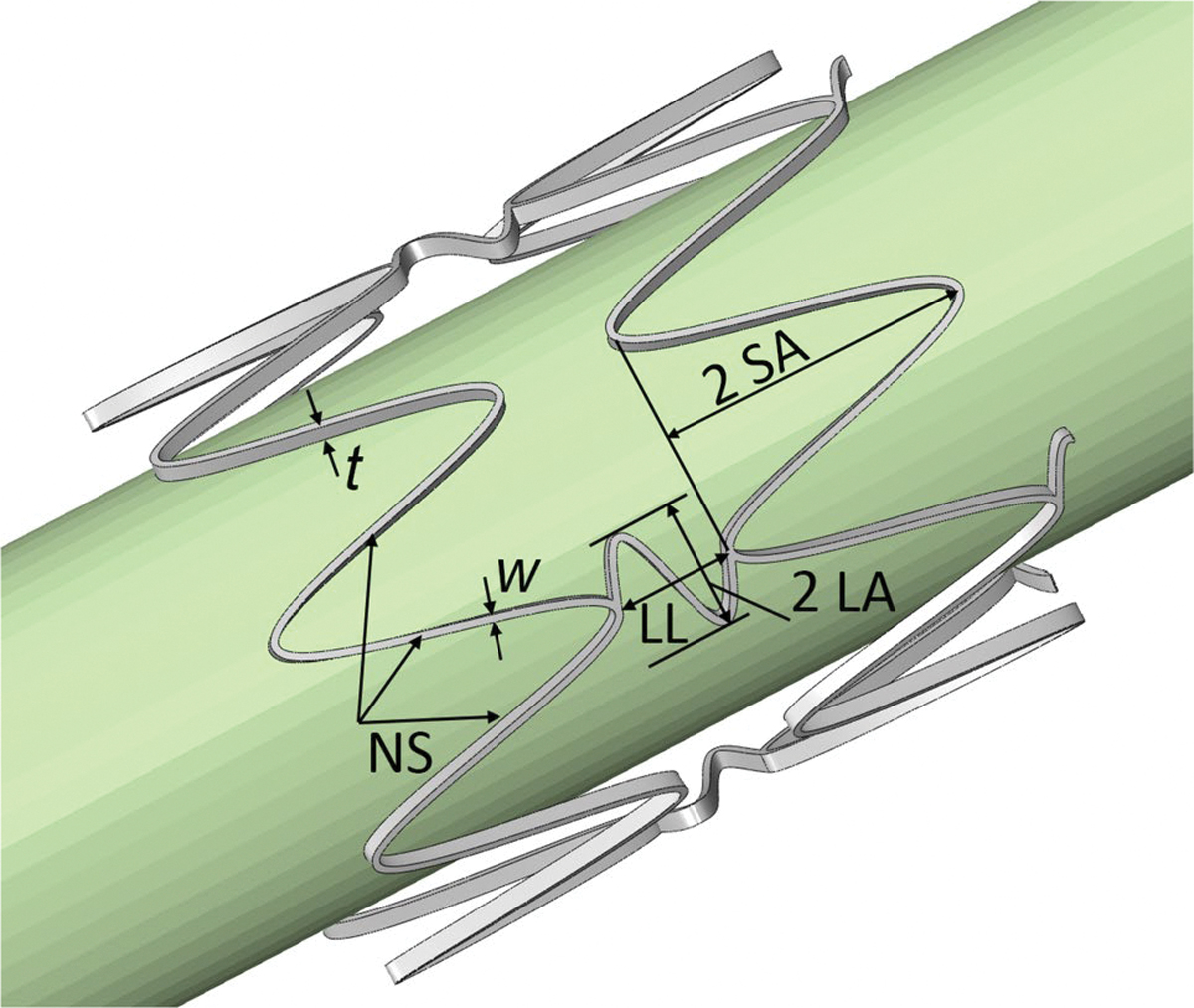
Stent parameterization included t (strut thickness), w (strut width), NS (number of struts), LA (link amplitude), SA (strut amplitude), and LL (link length)

Evaluation of commercial PAD stents has shown that sharp peaks formed at the junctions of stent struts in a crown configuration contribute to arterial abrasion during cyclic deformations [36]. To mitigate this issue, our stent design incorporates sine-wave rings connected by sine-wave links of a rectangular cross-section (Fig. 1). The stent’s Computer-Aided Design geometry was parametrized as an equation-driven spline for both rings and links and modeled in Solid-Works 2022 as a flat, unwrapped sheet. Five parameters were considered for the optimization study: strut thickness (t), strut width (w), number of struts (NS), link amplitude (LA), and strut amplitude (SA). The average nominal diameter of the human femoropopliteal artery at the AH (the area that experiences severe deformations during limb flexion) is approximately 6–8 mm in most adults [53–55]. Based on a typical oversizing factor of 1 mm, stent’s outer diameter was set to 8 mm. This configuration resulted in seven rings and six sets of connecting links, with an overall link length of π/2 mm in all models. To enhance flexibility, the links were arranged to connect every other peak of the sine-wave structure.

A sequential approach was employed for optimizing stent configurations. The process began with a design of experiments (DOE) using the central composite design (CCD) method to reduce simulation costs while identifying the optimal stent configuration. Five independent stent parameters (t,w, NS, LA, and SA) were varied across three levels (mean, lower, and upper bounds) based on values derived from commercial stents [25, 34], as summarized in Table 1. This initial DOE produced 43 stent configurations with values summarized in Table 4.

### A Finite Element model of the Stent and the Artery

The 2D stent CAD model was imported into Abaqus/Explicit 2017, meshed with C3D8R linear hexahedral elements and C3D4 tetrahedral elements, and then wrapped into a tubular structure. The stent was modeled using a superelastic nitinol material, with material parameters adopted from Gökgöl et al. [49] and summarized in Table 2.

**Fig. 2:**
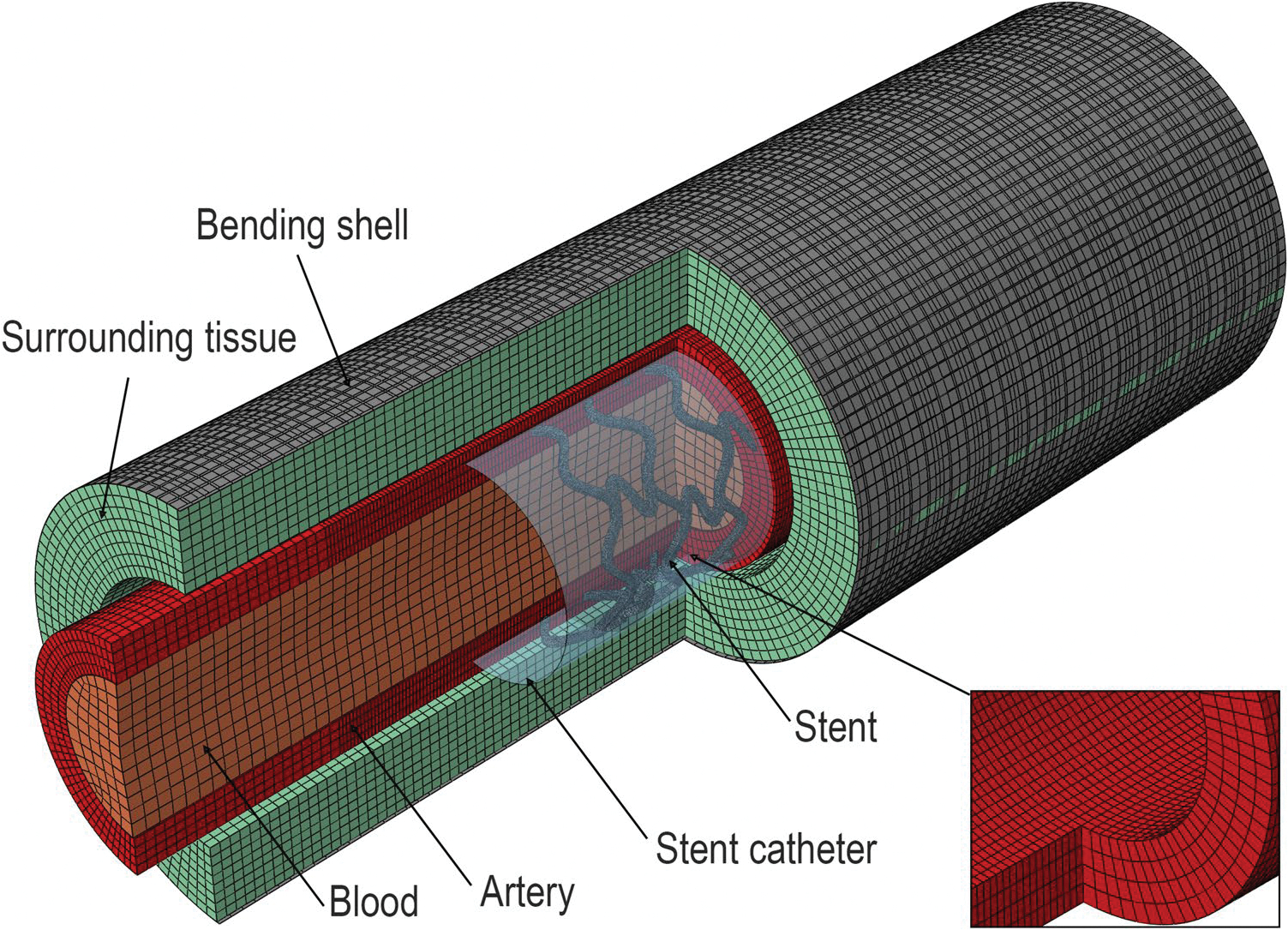
The finite element model of the femoropopliteal artery, showing the arterial segment with the surrounding tissues, as well as the stent and catheter used to crimp it. The insert shows mesh refinement in the center region of the artery

Each 3D stent design was then deployed and subjected to limb flexion-induced arterial deformations using finite element analysis (FEA). The model consisted of six components (Fig. 2): the artery wall, blood, compressible surrounding tissues, a rigid shell that applied deformations to the artery, the stent, and a catheter that crimped and deployed the stent. This modeling framework, including arterial geometry, anisotropic material properties, surrounding tissue support, and deformation boundary conditions, is based on experimentally measured human FPA biomechanics and on our previously validated FEA approaches [7, 28, 29, 56, 57], ensuring that the model represents a physiologically appropriate and scientifically supported representation of FPA mechanics.

Although stents are typically deployed in arteries containing plaque, plaque was not included in our analysis due to its highly variable size, shape, and structure, which would have significantly complicated the parametric analysis. At the same time, the addition of one generic plaque shape and properties would not add substantial value to the optimization in terms of generalizability to any type of plaque. Consequently, the angioplasty procedure was also excluded from the model since it is closely associated with the presence and characteristics of plaque.

The arterial segment represented the distal superficial femoral/proximal popliteal artery at the AH, as this region experiences severe deformations during limb flexion [27, 33] and is one of the most frequent sites for occlusive disease development [58]. The length of the arterial segment was set to three times the stent length to avoid edge effects. The artery was modeled as a tube with an inner diameter of 6 mm, a uniform thickness of 1.5 mm, and a length scaled by the inverse of the longitudinal prestretch, set at $\lambda_{z}\;=\;1.1$ [59]. A hyperelastic anisotropic two-fiber Holzapfel-Gasser-Ogden model [60] was used to represent the mechanical properties of a 60–70-year-old artery. The material parameters were set as follows: $C_{0}=23.3\;kPa,\;C_{1}^{\mathrm{col}}=12.04\;kPa,\;C_{2}^{\mathrm{col}}=18.2,\;\gamma =45.22^{\circ }$. These values reflect the typical mechanical behavior of femoropopliteal arteries in this age group [55, 56, 61]. The density of the artery was set to 1 g/cm^3^. Following the mesh sensitivity analysis, the artery was meshed with 102,720 linear hexahedral elements of type C3D8R to ensure the accuracy of mean intramural stresses.

The artery was filled with a soft, nearly incompressible material to simulate the supportive effect of blood on the arterial wall during deformations, following a previously established methodology [29]. This approach avoided the additional computational expense and complexity of fluid-structure interactions while still providing adequate support during bending. The supporting body, representing blood, was modeled as a linear elastic material with a Poisson ratio of 0.499 and a Young’s modulus of 35 kPa. The Young’s modulus was chosen to achieve a target arterial pinching ratio of 1.41, measured in the unstented artery during our perfused cadaver studies [29]. Pinching was defined as the ratio of the largest to the smallest diameter of the arterial cross-section. The structure representing blood was meshed with 12,596 linear hexahedral elements of type C3D8R. A mesh sensitivity analysis was not performed for this segment, as it primarily served as a supportive structure, and no specific quantities were calculated in this region.

The surrounding tissues were simulated using a compressible tube encasing the artery, with an inner diameter of 9.9 mm. This value was determined through an iterative procedure involving the application of axial compression and internal pressure to the artery, followed by recording the resulting outer diameter at the point of contact with the surrounding tissues. The thickness of the tube representing the surrounding tissues was set at 3.1 mm, with a length of 71.96 mm. The surrounding tissue was modeled as a neo-Hookean hyperelastic material with a density of 1 g/cm^3^, D_1_ = 36, and C_10_ = 20.8 kPa. These values were selected to enable physiologically realistic arterial bending while permitting vessel expansion during stent deployment. The surrounding tissue domain was meshed with 146,880 linear hexahedral elements of type C3D8R.

### Simulation of Stented Artery Deformations

**Fig. 3:**
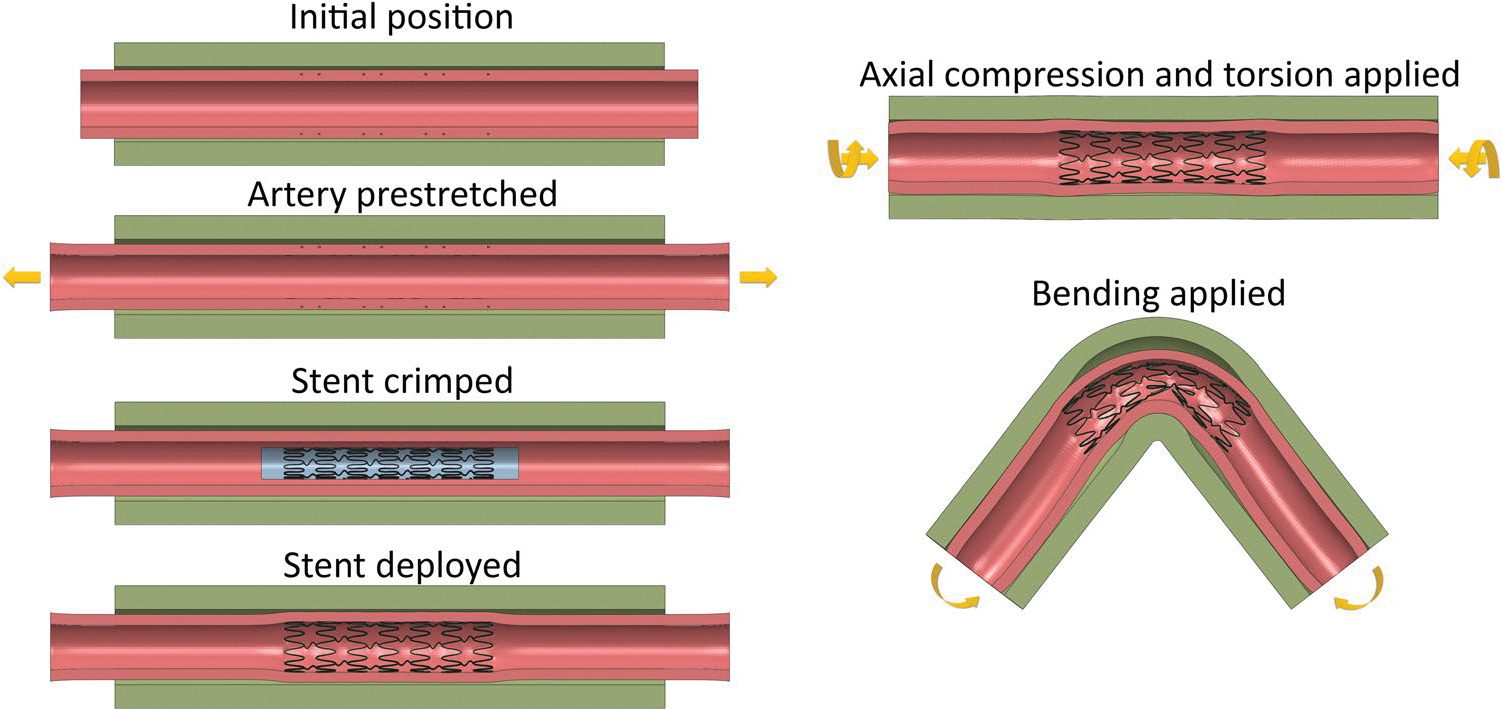
Simulation steps performed for each model, including arterial pre-stretch, stent crimping and deployment, and bending of the stented artery from a straight configuration to the deformed configuration corresponding to acute 60° limb flexion at the knee, i.e., gardening / fetal position

Arterial deformations were applied using a guiding shell, modeled as a stiff cylindrical shell surrounding the tissues, with a diameter of 16 mm. It was meshed with SFM3D4R surface elements and assigned a density of 6.5 g/cm^3^. The simulation began by stretching the artery to its *in situ* length ($\lambda_{z}=1.1$) and pressurizing it to 100 mmHg. During this step, no-penetration contact was enforced for all components, except for the stent and stent catheter. The stent was then radially crimped to an outer diameter of 4 mm by applying radial displacement to the stent catheter and subsequently deployed within the artery. Stent-artery interaction was activated during the deployment phase. In the next step, twisting and compression were applied to the ends of the artery (Fig. 3). A twisting rate of 16°/cm and 19% axial compression of the stretched length were applied, based on the most typical twist and compression observed at the AH during the most acute 60° limb flexion at the knee [7, 28], corresponding to the gardening / fetal position. Bending deformations were applied in three intermittent steps by displacing the nodes of the guiding shell to achieve a bending curve with a 9 mm radius of curvature, reflecting typical AH bending in the gardening / fetal position [7]. The guiding shell transferred the bending deformation to the artery through the compressible surrounding tissue. Variable mass scaling was used to reduce simulation time. All contacts were defined as no-penetration surface-to-surface contacts enforced through the penalty method. A friction coefficient of 0.4 was applied to all contact interfaces, except for the stent-artery interface, which used a friction coefficient of 1.0. Step increments and step times were adjusted to keep the kinetic energy within 5–10% of the internal energy to ensure quasi-static loading.

### Multi-Objective Optimization

Stents were optimized based on four objectives: (1) maximizing stent-artery apposition, (2) reducing pinching, (3) minimizing mean intramural stress, and (4) minimizing the arterial high-stress (>100 kPa) area. These objectives were selected to promote vessel patency (objectives 1 and 2) and minimize vascular injury (objectives 3 and 4), thereby reducing the risk of restenosis [62].

Several key endpoints were obtained after each simulation and used in the optimization process. Intramural Von Mises stresses were evaluated along the stented region of the artery and in the adjacent non-stented regions, extending to half the stent length on either side. A finer mesh was applied in this region (Fig. 2) to ensure accurate stress calculations, and the mean intramural stress was computed. The luminal area subjected to stresses above 100 kPa was also measured and normalized to the total luminal area. The 100 kPa threshold was selected based on data from Anttila et al., which indicated that damage accumulation starts to occur in 61- to 70-year-old femoropopliteal arteries at this stress level [52]. Stent-artery apposition was assessed both qualitatively and quantitatively, with the quantitative measure defined as the percentage of the stent strut area in contact with the arterial wall. Finally, arterial pinching at the midpoint of the artery was calculated as the ratio of the larger diameter to the smaller diameter, where a ratio of 1.0 corresponds to a perfectly circular lumen.

The response surface methodology (RSM) was used to construct models describing how dependent response variables were influenced by independent variables (t,w, NS, LA, and SA). Each response variable (mean intramural stress, proportion of the artery with Von Mises stresses exceeding 100 kPa, stent-artery apposition, and pinching) was fitted with a second-order polynomial model (Eq. 1). In this model, y represents the response, while $\beta_{0},\;\beta_{i},\;\beta_{ii}$, and $\beta_{ij}$ are the coefficients for the constant, linear, quadratic, and interaction terms, respectively; $x_{i}$ are the independent variables (k=5*)*, and ε represents the error term [63]. The optimal values of the independent variables were then determined using multi-response optimization.

(1) $$y=\beta_{0}+\sum_{i=1}^{k}\beta_{i}x_{i}+\sum_{i=1}^{k}\beta_{ii}x_{i}^{2}+\sum_{i=1}^{k}\sum_{i\neq j=1}^{k}\beta_{ij}x_{i}x_{j}+\varepsilon$$

Multi-objective optimization was achieved using the desirability function approach. Briefly, this method involves transforming each response into an individual desirability function, linking them to a utility function ranging from 0 to 1. When a response reaches its target value, the desirability function attains a value of 1. The responses are categorized into three main types: nominal-the-best (NTB), smaller-the-best (STB), and larger-the-best (LTB). In this study, the objective function aimed to minimize mean arterial stress, luminal area with stress over 100 kPa (high-stress area), and pinching, while maximizing stent-artery apposition. Therefore, STB and LTB types of desirability functions were used.

The desirability functions $d_{j}$ were defined as follows, with Eq. 2 for maximizing a response and Eq. 3 for minimizing a response, according to methods established previously [64, 65].

Maximizing a response (LTB):

(2) $$d_{j}(x)=\left\{\begin{array}{c} 0\;\text{if}\;{\hat{y}}_{j}(x)\leq y_{j}^{min}\\ \left(\frac{{\hat{y}}_{j}(x)-y_{j}^{min}}{y_{j}^{max}-y_{j}^{min}}\right)\;\text{if}\;y_{j}^{min}\leq {\hat{y}}_{j}(x)\leq y_{j}^{max}\\ 1\;\text{if}\;{\hat{y}}_{j}(x)\geq y_{j}^{max} \end{array}\right.$$

Minimizing a response (STB):

(3) $$d_{j}(x)=\left\{\begin{array}{c} 1\;\text{if}\;{\hat{y}}_{j}(x)\leq y_{j}^{min}\\ \left(\frac{y_{j}^{max}-{\hat{y}}_{j}(x)}{y_{j}^{max}-y_{j}^{min}}\right)\;\text{if}\;y_{j}^{min}\leq {\hat{y}}_{j}(x)\leq y_{j}^{max}\\ 0\;\text{if}\;{\hat{y}}_{j}(x)\geq y_{j}^{max} \end{array}\right.$$

Once the individual desirability functions were established, a composite desirability function D(x) was constructed as a product of individual desirability functions (Eq. 4). Since the relative importance of response variables in determining overall stent performance is unclear, all response variables were assigned equal weight.

(4) $$D(x)=\prod_{j=1}^{m}d_{j}(x)$$

In these equations, $y_{j}^{\min}$ and $y_{j}^{\max}$ represent the minimum and maximum values for the response ${\hat{y}}_{j}$, respectively; x are independent variables, and m=4 responses.

Once a set of optimal input variables was determined, CAD model of the optimized stent was built, and FEA simulation of stent deployment and arterial deformation was performed to obtain the actual values of response variables. In addition, stent fatigue performance was evaluated by calculating strain amplitude and mean strain in every element between the fully deformed and straight artery configurations.

### Statistical Analysis

Correlations between variables - t,w, NS, LA, and SA - were assessed using Pearson’s correlation coefficient. The normality of the data was evaluated with the Shapiro-Wilk test, while homogeneity of variances was assessed using Levene’s test. For each objective, the significance of each term in the RMS regression equation (Eq. 1) was evaluated using a two-tailed t-test with a significance level of 0.05. The strength and direction of the association between a model term and the response variable were described by the corresponding regression coefficient.

## Results

### Stent-Artery Interactions

**Fig. 4:**
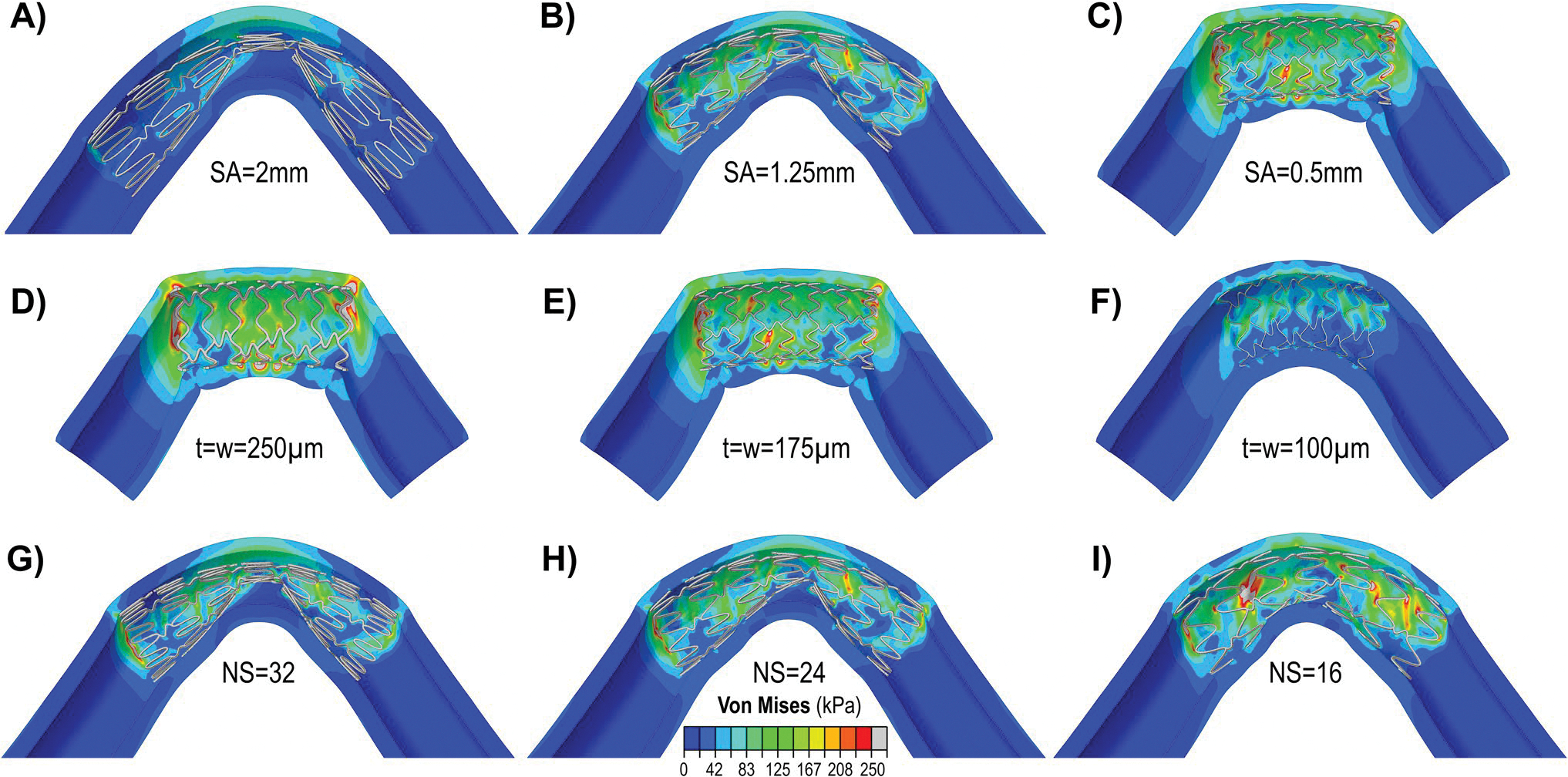
Von Mises (kPa) stress maps demonstrating the effects of strut amplitude (SA: A, B, C), strut thickness (t) and width (w) (D, E, F), and the number of struts (NS: G, H, I) on stent-artery interaction during combined loading. In panels **A**, **B**, and **C** w=175μm,t=175μm, and NS = 24; in panels **D**, **E**, and **F** SA = 0.5; in panels **G**, **H**, and **I** w=175μm,t=175μm, and SA = 1.25 mm

The interactions between the stent and the artery during combined loading were strongly influenced by the stent design. Figure 4 illustrates the effects of SA, t,w, and NS. The LA had no significant effect on the outcome variables. A longitudinal cut was made in the model to improve the visualization of stent-artery interactions.

The influence of SA on arterial stress, stent-artery apposition, and pinching is demonstrated in Fig. 4A–C for three different SA values, with w and t fixed at 175 μm and the NS set at 24. In the simulations, the LA was set to 0.4 mm for SA values of 2 mm and 0.5 mm, and to 0.8 mm for an SA of 1.25 mm. All stents contained 7 rings, but due to differences in SA, the overall lengths of the stents - and consequently the arteries (which were set to three times the stent length) - varied across the models. The results indicate that arteries treated with endovascular stenting featuring higher SA values experienced lower stresses and accommodated bending more effectively; however, they exhibited poorer stent-artery apposition (Fig. 4A). In contrast, lower SA values resulted in improved stent-artery apposition, with a more pronounced luminal circularity, but also produced higher stresses (Fig. 4C).

The second and third most influential design parameters were w and t. Their effects, for struts with a square cross-section (i.e., t=w), are illustrated in Fig. 4D–F for stents with SA of 0.5 mm. In panels Fig. 4D and F the NS and LA were set to 16 and 0.8 mm, respectively, while in Fig. 4E, they were set to 24 and 0.4 mm. Stents with a small SA and a large strut cross-section produced significant stress concentrations and biomechanical disruptions at the ends of the stented segment (Fig. 4D). In contrast, stents with smaller cross-sections accommodated bending, twisting, and compression more effectively, resulting in improved stent-artery apposition and lower, more uniformly distributed arterial stresses (Fig. 4F). While a larger cross-section slightly improved the luminal circularity, it significantly increased the mean arterial wall stresses. For example, a stent with a w=t=250μm enhanced the luminal circularity 12% compared to a stent with w=t=100μm; however, the arterial wall stress increased 147%. The improved stress distribution achieved with a reduced strut cross-section also eliminated areas of concentrated stress at the stent ends.

The effect of NS on the arterial stress, stent-artery apposition, and pinching is illustrated in Fig. 4G–I. In these simulations, both w and t were set to 175 μm, and the SA was set to 1.25 mm. The results demonstrate that arterial wall stress increased as the NS decreased. However, this increase in stress was due to improved stent-artery apposition. When the NS was high, fewer struts made contact with the arterial surface, reducing overall stress but also weakening the structural support provided by the stent and resulting in stent instability and buckling, potentially compromising flow.

### Significance of Design Parameters in Stent Performance

**Fig. 5:**
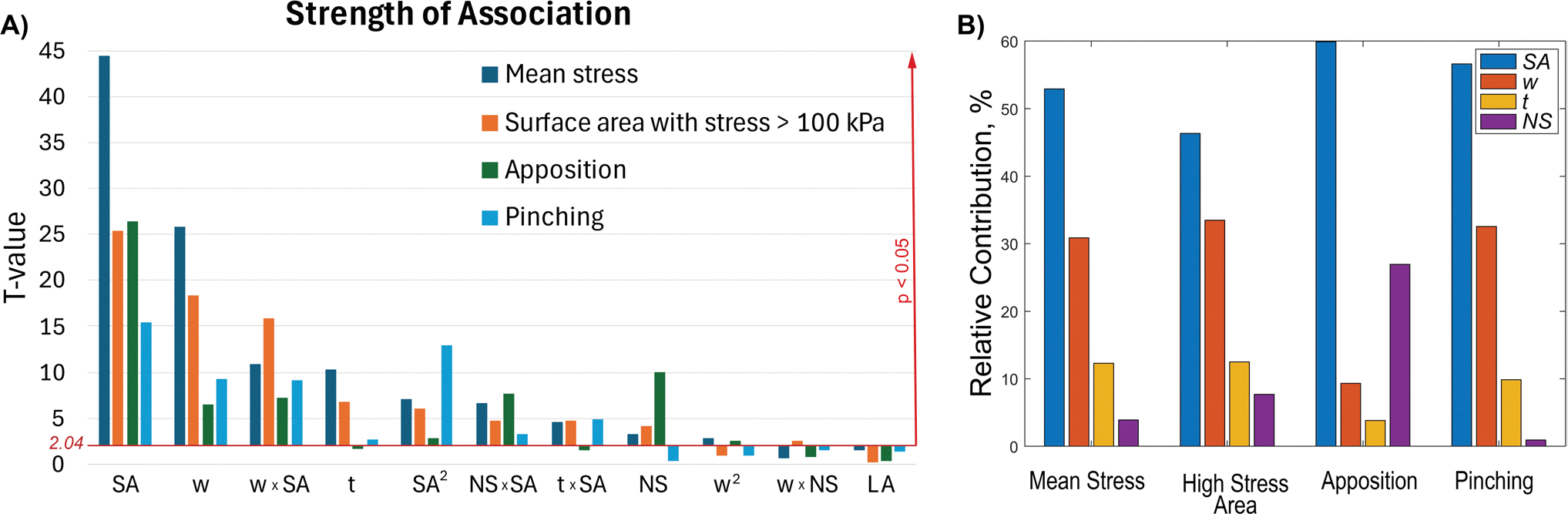
**A** Factors significantly affecting stent performance in terms mean intramural stress, proportion of the artery surface area with stress exceeding 100 kPa, stent-artery apposition, and vessel lumen pinching. The stronger the association with the stent design parameter and output variable, the taller the bar. Bars that are below the red horizontal line ($t_{crit}\;=\;2.04,\;p\;=\;0.05$) denote non-significant association. **B** Relative contribution of each parameter on the mean stress, surface area with stress over 100 kPa, apposition, and pinching, evaluated at the midpoint of the factors

The *p*-values were used to evaluate the significance of linear, quadratic, and interaction coefficients, where a lower *p*-value indicates that the corresponding coefficient is more significant, meaning that the selected factor strongly influences the desired stent behavior. In this study, the critical *t*-value for a significance level o*f* 0.05 was 2.04. The analysis of the calculated coefficients and their respective t-values showed that among the design factors, w,t, NS, SA, and their interaction terms $w^{2}$, ${\mathrm{SA}}^{2}$, w×SA, NS×SA, t×SA,w×NS, were significant (Fig. 5A). SA had the largest effect on all response variables, affecting intramural stresses, stent-artery apposition, and vessel pinching. It was followed, in order of significance, by w,t, and NS, while LA had no significant effect on stent performance.

Retaining only the significant contributors, the second-order polynomial models from Eq.1 simplify to the reduced forms shown in Eq. 5, with coefficients β summarized in Table 3. These models accurately predict key outcomes (y) – including mean intramural stress, the proportion of luminal area with stress exceeding 100 kPa, stent-artery apposition, and pinching - with R^2^ values exceeding 0.96 for all variables.

(5) $$Y=\beta_{0}\;+\beta_{w}\cdot w+\beta_{t}\cdot t+\beta_{NS}\cdot NS+\beta_{SA}\cdot SA\;\;+\beta_{w^{2}}\cdot w^{2}+\beta_{SA^{2}}\cdot SA^{2}+\beta_{w\cdot NS}\cdot w\cdot NS\;\;+\beta_{w\cdot SA}\cdot w\cdot SA+\beta_{t\cdot SA}\cdot t\cdot SA+\beta_{NS\cdot SA}\cdot NS\cdot SA$$

To elucidate the influence of each stent design parameter (SA, w, t, and NS) on the model outcomes, a numerical sensitivity analysis was performed using the regression models described in Eq. 5. For each response variable, partial derivatives with respect to each design parameter were computed and evaluated at the midpoint of the corresponding parameter range. The resulting sensitivities were then normalized by their respective parameter ranges to enable comparison across variables with different units and scales. The relative contribution of each factor was expressed as the percentage of its normalized sensitivity relative to the total normalized sensitivity of all factors. These results are summarized in Fig. 5B for all four outcome measures.

Pairwise combinations of response surfaces for the significant interaction factors are presented in Appendix Figs. 7–10, with all other factors held at their upper, mid, or lower bounds. Due to the similarity in response surfaces for the mean intramural stress and the proportion of the artery experiencing stress above 100 kPa, only the response surfaces for the mean intramural stress are shown. The results indicate that increasing w,t, and NS leads to higher arterial stresses. Additionally, intramural stresses increased as SA decreased, with the effects of w,t, and NS becoming more pronounced at lower strut amplitudes (Fig. 7). Stent-artery apposition improved as the SA decreased from 2.0 mm to 0.5 mm, and this improvement was more substantial when the NS was high and w was low (Fig. 8). Pinching worsened as the SA increased from 0.5 mm to 1.25 mm, but improved at 2.0 mm (Fig. 9). This non-linear behavior likely resulted from the competing effects of apposition and arterial deformation. At the lower bound (SA=0.5mm), the stent maintained excellent apposition and conformed closely to the arterial wall, thereby minimizing luminal distortion. At the upper bound (SA=2.0mm), the stent provided limited circumferential support, and the artery tended to deform toward an oval shape, yielding moderate pinching values. At the midpoint (SA=1.25mm), apposition improved relative to the upper bound, but the asymmetric displacement of the lumen - predominantly in one direction - produced greater apparent pinching (Fig. 10). Table 4 summarizes the central composite design matrix, listing all combinations of design variables and their corresponding responses for each DOE experiment.

The optimal values for all input parameters were determined using the desirability function methodology, aiming for complete stent-artery apposition, minimal pinching (targeting a circular cross-section with a ratio of 1.0), zero proportion of the artery experiencing Von Mises stress exceeding 100 kPa, and the lowest possible mean intramural stress.

The optimal values for the five design parameters (w,t, SA, NS, LA) were found to be 100 μm, 100 μm, 0.5 mm, 16, and 0.8 mm, respectively. By substituting these optimized values into the regression models, the predicted responses for the mean intramural stress, the proportion of the artery with stress exceeding 100 kPa, stent-artery apposition, and pinching were computed and verified against numerical results. The RSM regression models predicted values of 98.0% for stent-artery apposition, 0.1% for the proportion of the artery with von Mises stress exceeding 100 kPa, 28.5 kPa for mean arterial wall stress, and 1.18 for pinching. These predictions were consistent with the numerical simulation results obtained using the optimized parameters, which yielded 98.1%, 0.4%, 28.9 kPa, and 1.15, respectively.

Although the current RSM optimization produced a stent configuration that met the performance objectives, the optimized design leaned toward the lower bounds for w,t, SA, and the NS, while favoring the upper bound for LA. This observation suggested the potential for further performance improvements by using even smaller w,t, and SA. To evaluate this possibility, the optimization process was revisited, and a second DOE was conducted. Guided by insights from the first DOE, which showed that LA had an insignificant effect on overall stent performance, LA was held constant at 0.4 mm in the new design space. Similarly, NS was fixed at 16 to limit computational cost, as it was found to be less influential compared to w,t, and SA. The lower, middle, and upper bounds were defined as 50, 100, 150 μm for both w and t, and 0.3, 0.5, 0.7 mm for SA. In addition, a crimpability constraint was introduced – requiring that the optimized configuration remain mechanically stable during crimping without buckling – to ensure manufacturability and deployment feasibility. The results of the second DOE are summarized in Table 5.

**Fig. 6:**
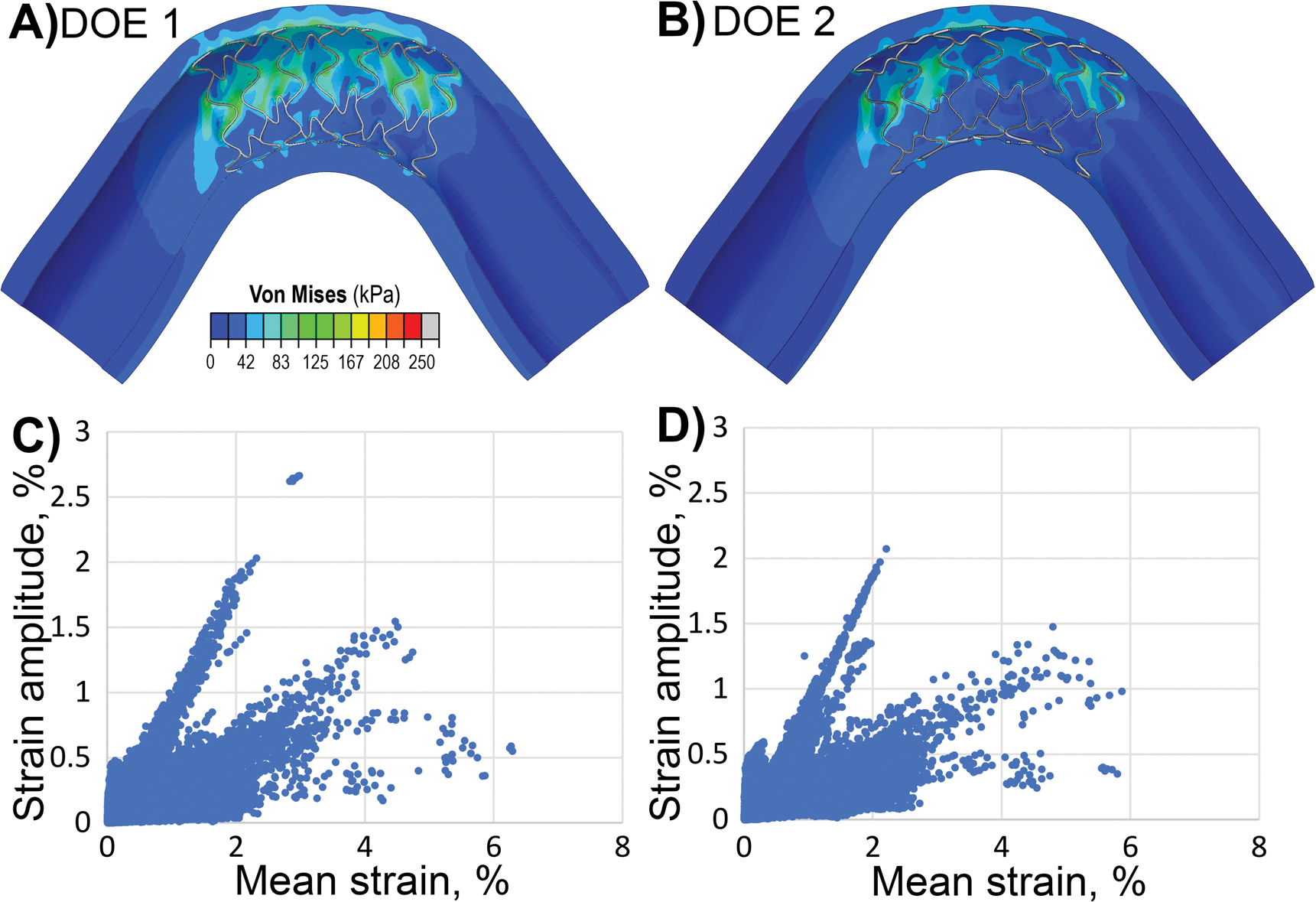
**A**, **B** Von Mises stress (kPa) maps for the optimized stent design obtained with **A** the first DOE and **B** the second DOE. **C**, **D** Fatigue performance of optimized stents during limb flexion depicting a point cloud of strain amplitude plotted versus mean strain for each stent element for the C) first and D) second DOE

**Fig. 7:**
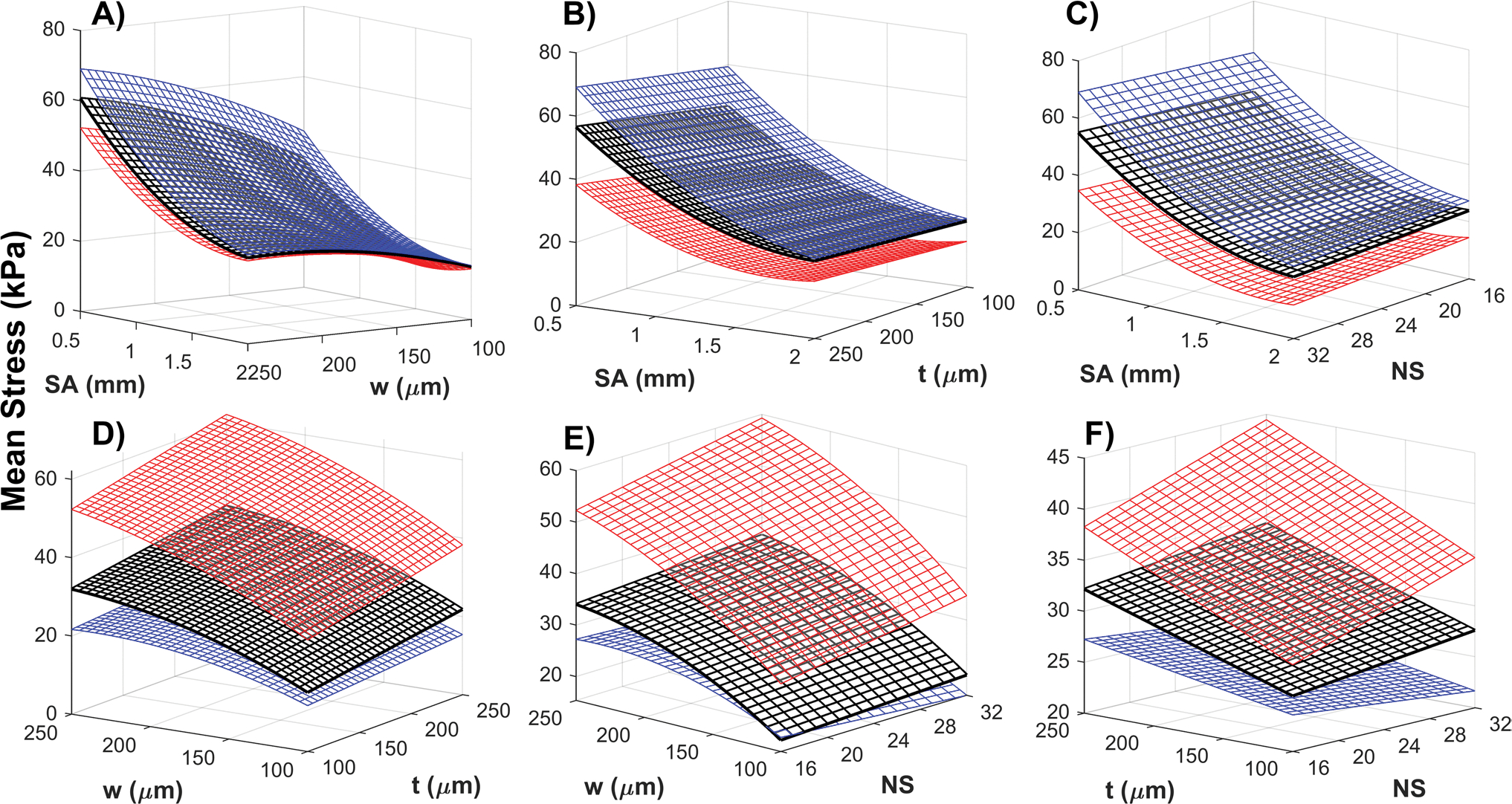
Response surface plots for the average arterial wall stress (kPa). Evaluated parameters include: **A** strut amplitude-width, **B** strut amplitude-thickness, **C** strut amplitude-strut number, **D** width-thickness, **E** width-strut numbers, **F** thickness-strut number. Blue, black, and red surfaces are evaluated at the upper, mid, and lower bounds of the remaining two parameters in each panel

**Fig. 8:**
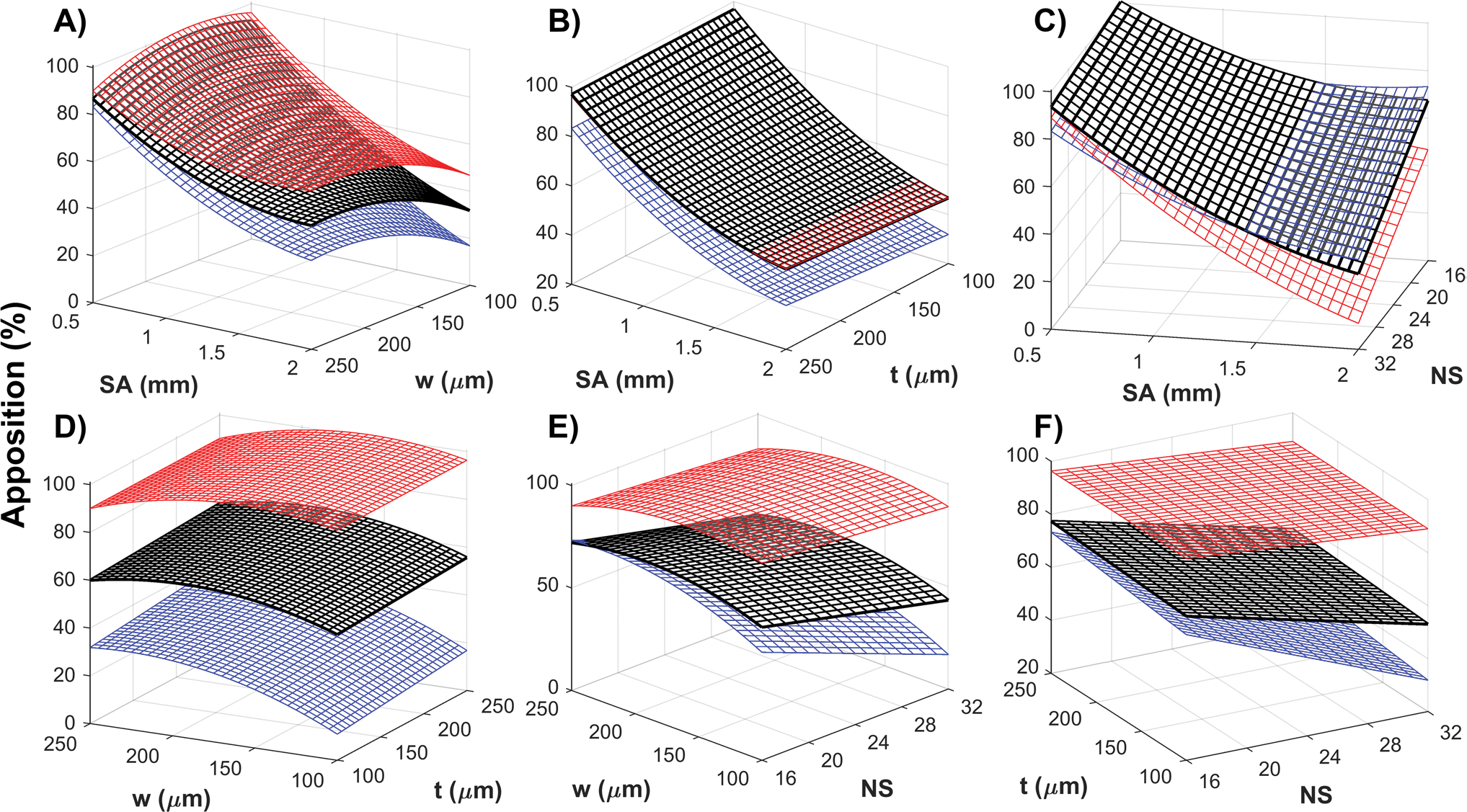
Response surface plots for stent-artery apposition (%). Evaluated parameters include: **A** strut amplitude-width, **B** strut amplitude-thickness, **C** strut amplitude-strut number, **D** width-thickness, **E** width-strut number, **F** thickness-strut number. Blue, black, and red surfaces are evaluated at the upper, mid, and lower bounds of the remaining two parameters in each panel

**Fig. 9:**
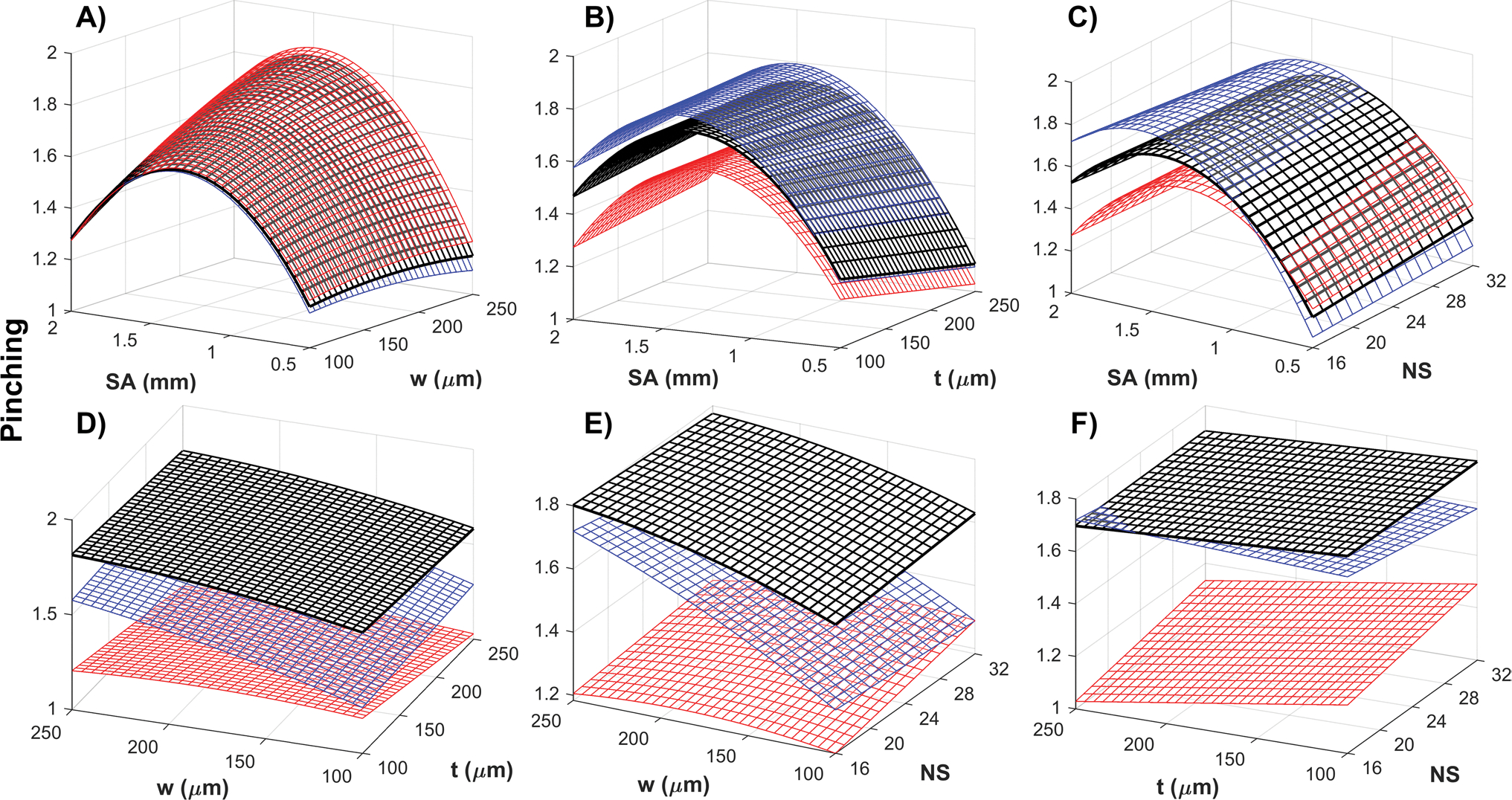
Response surface plots for the arterial pinching. Evaluated parameters include: **a** strut amplitude-width, **b** strut amplitude-thickness, **c** strut amplitude-strut number, **d** width-thickness, **e** width-strut number, **f** thickness-strut number. Blue, black, and red surfaces are evaluated at the upper, mid, and lower bounds of the remaining two parameters in each panel

**Fig. 10:**
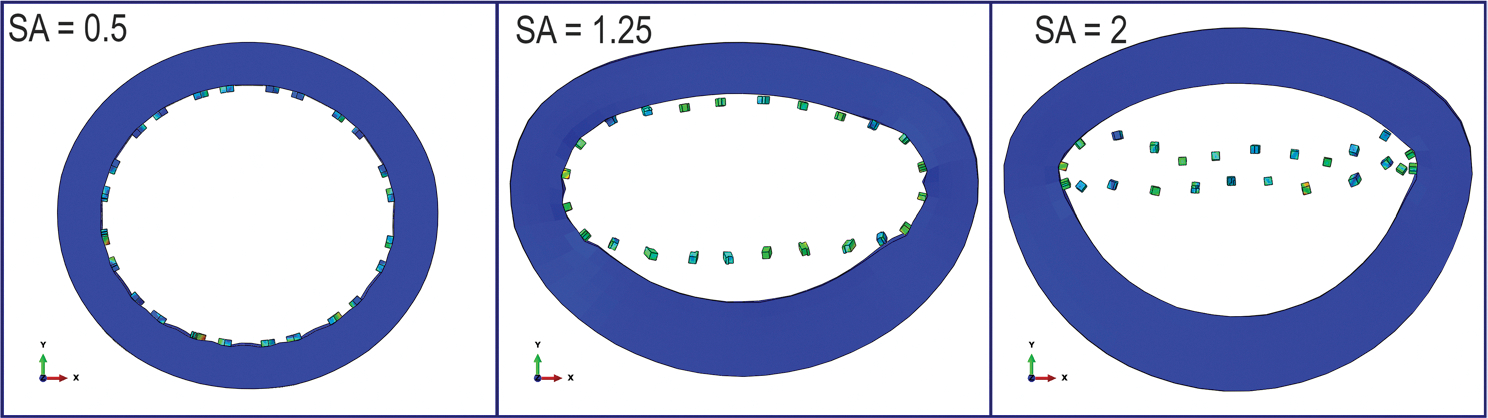
Effects of strut amplitude (SA) on the cross-sectional shape of the artery at its midpoint

Using the second DOE, a new optimized configuration was determined to be 70 μm for w, 140 μm for t, 0.5 mm for SA (Fig. 6B). By inputting these optimized values into the regression models, the predicted responses for the mean intramural stress, the proportion of the artery with Von Mises stress exceeding 100 kPa, stent-artery apposition, and pinching were calculated and verified against the numerical results. The RSM regression models predicted 96.1% for stent-artery apposition, 0.2% for the proportion of the artery with stress exceeding 100 kPa, 25.5 kPa for the mean intramural stress, and 1.11 for pinching. These predictions closely aligned with the numerical simulation results, which yielded 96.4% for stent-artery apposition, 0.1% for the proportion of the artery with stress exceeding 100 kPa, 25.23 kPa for the mean intramural stress, and 1.13 for pinching. Both optimized configurations, from DOE1 and DOE2, together with their respective response values, are summarized in Table 6.

Figure 6C–D illustrates the fatigue performance of the optimized stents during limb flexion, shown as a point cloud of strain amplitude plotted versus mean strain for each stent element. Most elements exhibited mean strains below 2% and strain amplitudes under 0.4%, with 97.3% and 97.7% of elements meeting this criterion in the first and second DOE, respectively. Only 0.3% (first DOE) and 0.15% (second DOE) of elements experienced strain amplitudes above 1%, while a negligible fraction −0.006% and 0.0005%, respectively - exceeded 2%.

## Discussion

The femoropopliteal artery undergoes severe axial compression, bending, twisting, and pinching during limb flexion [7, 27, 30–33], with the greatest deformations occurring at the AH and behind the knee. These localized deformations are strongly associated with disease development [58], stent fractures [45, 58, 66], and the high failure rates of PAD endovascular treatments [33]. Studies using intra-arterial markers in perfused cadaver models [7, 28] have revealed the true extent of these deformations, demonstrating that they are significantly larger and more localized than previously recognized [27, 33]. Subsequent bench-top [34] and cadaver experiments [35] evaluating PAD stents under these deformations showed that many existing stents fail to accommodate them adequately, resulting in buckling, diameter pinching, restricted and exacerbated deformations both within and beyond the stented segment, and vessel wall abrasions [36], caused by the poor biomechanical compatibility of stiff stents with limb flexion-induced arterial deformations. To address these challenges, we employed a multi-objective optimization approach using response surface methodology – a powerful optimization technique for complex relationships between input factors and output responses. This approach enabled the identification of optimal stent design parameters while providing insights into the interactions between design factors and their effects on stent performance.

Our results demonstrate that stent design features significantly influence stent-artery interactions. Intramural stress resulting from these interactions was positively associated with strut width, thickness, and the number of struts, while strut amplitude showed a negative association with arterial stress. Link strut amplitude, however, did not exhibit a significant effect. Among the design parameters, strut amplitude had the strongest influence on stent performance. As strut amplitude decreased, arterial stress increased significantly, and this effect was amplified by higher strut width, thickness, and the number of struts. At lower strut amplitudes, the impact of width, thickness, and the number of struts on arterial stress became more pronounced. Pinching worsened as strut amplitude increased, and stent-artery apposition improved as the strut amplitude decreased, with this enhancement being more pronounced at higher numbers of struts and smaller strut widths. Notably, an increase in strut amplitude or a reduction in strut width and thickness decreased the structural stiffness of the stent, resulting in reduced arterial expansion.

The width and thickness of stent struts strongly influenced stent performance, with higher values associated with increased arterial stress. Among these two parameters, the impact of width was more pronounced than that of thickness. The effect of width and thickness on arterial stress was also dependent on other design parameters, particularly strut amplitude, with a greater influence observed at lower strut amplitudes. Variations in strut width and thickness significantly affected pinching, consistent with previous findings [67]. An increase in strut thickness elevated the chronic outward force (COF), though this did not necessarily reduce vessel pinching. High COF is linked to higher rates of restenosis due to persistent vessel injury caused by self-expanding nitinol stents [68, 69]. Conversely, reducing strut width and thickness decreased stent stiffness, improving performance under bending [67]. However, this also increased arterial pinching due to the stent’s reduced ability to maintain an open lumen. Balancing width and thickness is therefore critical to achieving sufficient COF while maintaining acceptable arterial stress. Stent-artery apposition improved significantly with increased strut width, particularly at higher strut amplitudes, whereas thickness had little effect on apposition. Interestingly, thickness and width had different effects on vessel pinching: increasing thickness produced a linear reduction in pinching, while increasing width led to a non-linear reduction. The effects of thickness and width on pinching were also contingent on strut amplitude. At SA=2, pinching improved as thickness decreased, whereas at SA=0.5, pinching improved as thickness increased.

The number of struts also had a significant influence on arterial stress, the area of the artery with stress exceeding 100 kPa, and stent-artery apposition, although it did not notably affect pinching. Contrary to the findings of Azaouzi et al [67], who suggested the limited significance of the number of struts in the circumferential direction, our results indicate a substantial impact. This effect was particularly evident when combined with strut thickness, as an increase in the number of struts elevated arterial stress, regardless of the strut thickness value. The influence of the number of struts on stent-artery apposition was highly dependent on strut amplitude. In general, arterial stress increased with a higher number of struts; however, this relationship was heavily influenced by stent-artery apposition. In cases of good apposition, a larger surface area of the stent exerting outward force on the arterial surface led to an increase in arterial stress, even when the number of struts was reduced. The effect of the number of struts on arterial stress was also contingent on strut amplitude. At SA=0.5, arterial stress increased with a higher number of struts, whereas at SA=2, stress decreased as the number of struts increased.

When the stent failed to accommodate the combined deformations of the artery, stress localization predominantly occurred at the ends of the stented region. Thinner struts and higher strut amplitudes improved the accommodation of arterial deformations and reduced stress localization in these areas. However, higher strut amplitudes and increased strut numbers led to greater pinching and poorer stent-artery apposition. These findings highlight the inherent tradeoffs between achieving different objectives: improving stent-artery apposition and reducing pinching often resulted in larger arterial deformations proximal and distal to the stented segment, accompanied by higher arterial wall stress. Using RSM, it was possible to minimize overall arterial stresses and stress localization by optimizing the stent’s geometrical design while maintaining acceptable stent-artery apposition and low pinching. Our results suggest that stents with smaller cross-section struts, lower strut amplitudes, and fewer struts exhibit better stent-artery apposition and reduced pinching. Although not explicitly analyzed in this study, such stent geometries are also likely to promote improved flow patterns, reducing the risk of neointimal hyperplasia [70]. Prior studies [71] have shown that increasing strut amplitude reduces radial force, while greater strut width and thickness increase it. These findings align with our results, where stent-artery apposition improved with greater strut width and thickness but significantly decreased with higher strut amplitudes. Additionally, other studies [72] have demonstrated that using blunted strut corners and higher amplitudes decreases arterial wall stresses, which is consistent with our observations that lower arterial stresses were associated with higher strut amplitudes. While our findings support the benefits of reducing strut cross-section - a conclusion consistent with prior studies linking a lower metal-to-artery ratio with better clinical outcomes [73] - this reduction must be balanced with the strength and fatigue performance of the stent to ensure long-term durability. Fatigue evaluation is important in order to select an appropriate type of nitinol. Purity of the nitinol is strongly associated with its fatigue performance, and fatigue safety limits vary greatly from 0.4% for the standard, generation I nitinol to 2% for the pure, generation III nitinol. While over 97% of the stent elements in both optimized designs exhibited a strain amplitude of less than 0.4%, a small fraction of the elements (0.006% to 0.0005% for the first and second optimized designs) experienced over 2% strain amplitudes in our study, which suggests opting for newer generations of the nitinol would be advisable.

Our study aimed to design an optimized PAD stent compatible with the complex biomechanical deformations of the femoropopliteal artery during limb flexion, but the findings should be interpreted within the context of the study’s limitations. First, although computational analysis provides valuable insights, real-world validation is essential. This includes successful manufacturing of the proposed design, followed by systematic bench and preclinical testing. Bench-top experiments in physiologically realistic mock vessels or *ex vivo* arteries subjected to controlled bending and torsion can be used to quantitatively assess stent apposition, luminal pinching, and mechanical deformation under severe limb flexion. High-resolution imaging modalities, such as micro-CT or optical coherence tomography, can then be employed to directly compare the experimentally observed deformed geometries with the computational predictions, thereby verifying the fidelity of the finite element models. In addition, preclinical animal studies [74] are necessary to evaluate biological responses, including arterial injury, neointimal formation, and remodeling, linking mechanical optimization to restenosis outcomes and long-term vessel healing. Until these steps are completed, the current work should be viewed as an optimization framework with the potential to improve device performance but without yet providing definitive clinical evidence of benefit. For this reason, the equal weighting of the four mechanical objectives should be interpreted as a strategy to identify geometries that improve multiple determinants of mechanical compatibility, rather than as a direct predictor of clinical outcomes.

Second, the fidelity of our computational model could be enhanced by incorporating additional factors such as surrounding soft tissues, blood flow, atherosclerotic plaque, and the angioplasty procedure itself. These elements were excluded to reduce complexity, but could influence outcomes. For instance, calcified or fibrous plaques, their concentric versus eccentric geometry, and lesion lengths are likely to alter stent expansion and apposition and should be included in future analyses. Additionally, while we focused on the most severe, worst-case deformations in this study, examining a broader spectrum of boundary conditions would provide further insight into performance variability. Third, although our optimization objectives were based on clinically relevant metrics, their relative contributions to long-term stent performance remain uncertain, and future studies could integrate additional endpoints such as flow disturbances or fatigue life. Lastly, restenosis is a multifactorial process driven by mechanical, biological, and systemic factors acting synergistically. External influences such as smoking, diabetes, pharmacotherapy, and lifestyle may substantially affect device performance-sometimes to a greater extent than stent mechanics alone. Despite these limitations, our study represents a step forward in developing an optimized PAD stent tailored to the complex deformation environment of the femoropopliteal artery. By minimizing stress concentrations and improving mechanical compatibility, the proposed design has the potential to reduce arterial injury and restenosis. The validation strategies outlined above will be critical for translating this computational framework into a clinically viable device capable of improving long-term outcomes for patients undergoing endovascular PAD treatment.

## Figures and Tables

**Table 1:** The design space (mean, lower, and upper bounds) for each input parameter: *t* - strut thickness; *w* - strut width; *NS* - number of struts; *LSA* - link strut amplitude; *SA* - strut amplitude

Input variable	Lower bound	Medium value	Upper bound

Strut Thickness, *t* (μm)	100	175	250
Strut Width, *w* (μm)	100	175	250
Number of struts, NS	16	24	32
Link Amplitude, LA (mm)	0.0	0.4	0.8
Strut Amplitude, SA (mm)	0.50	1.25	2.00

**Table 2:** Nitinol material properties for the stent [49]

Austenite elasticity $E_{A}$ (MPA)	65000

Austenite Poisson’s ratio $\nu_{A}$	0.33
Martensite elasticity $E_{M}$ (MPa)	23500
Martensite Poisson’s ratio $\nu_{M}$	0.33
Transformation strain $\varepsilon^{L}$	0.046
Start of transformation loading $\sigma_{L}^{S}$(MPa)	465
End of transformation loading $\sigma_{L}^{E}$(MPa)	535
Start of transformation unloading $\sigma_{U}^{S}$ (MPa)	227
End of transformation unloading $\sigma_{U}^{E}$ (MPa)	187
Start of transformation stress in compression $\sigma_{CL}^{S}$ (MPa)	582
Volumetric transformation strain $\varepsilon_{V}^{L}$	0.046
Density, g/cm^3^	6.5

**Table 3:** Coefficients *β* for the second-order polynomial model in Eq. 5

Factor	*β*_0_	*β_w_*×10^−4^	*β_t_*× 10^−4^	*β_NS_*× 10^−3^	*β_SA_*× 10^−2^	*β*_*w*^2^_× 10^−6^	*β*_*SA* ^2^_	*β*_*w*•*NS*’_× 10^−5^	*β*_*w*•*SA*’_× 10^−5^	*β*_*t*•*SA*’_×10^−4^	*β*_*NS*•*SA*’_×10^−4^	*R* ^2^

Mean stress, kPa	−2.95	3732	788	542	−2649	−535	12.92	33	−6509	−272	−3725	0.99
High–stress area, %	−10.59	1045	304	122	−503	−80	5.19	67	−4425	−132	−1270	0.98
Apposition, %	103.30	3570	−115	135	−6440	−1313	14.45	13.2	11900	254	−11945	0.97
Pinching	0.36	15	−15	12	162.6	−5	−0.69	−2.6	168	9	−57.8	0.96

**Table 4:** The central composite matrix of independent variables and their response values

Exp. No.	Design variables					Response values			
		
	*w*, μm	*t*, μm	NS	LA, mm	SA, mm	Mean stress, kPa	High-stress area, %	Apposition, %	Pinching

1	175	175	24	0.4	2	21.41	0.06	59.15	1.53
2	250	250	32	0	0.5	71.52	17.29	93.32	1.07
3	175	175	24	0.8	1.25	29.93	1.61	76.80	1.73
4	100	250	16	0.8	2	15.57	0.01	53.63	1.31
5	100	100	16	0	2	14.54	0.00	45.42	1.30
6	100	250	16	0	0.5	39.31	3.97	97.68	1.08
7	100	100	32	0	2	14.19	0.00	14.64	1.29
8	250	250	32	0.8	0.5	69.64	18.27	94.01	1.07
9	250	250	16	0	2	29.03	1.27	82.37	1.79
10	250	100	32	0.8	2	19.42	0.11	35.28	1.51
11	100	250	16	0.8	0.5	36.86	2.96	98.05	1.08
12	250	100	16	0.8	2	22.80	0.25	67.48	1.61
13	250	100	32	0	0.5	60.75	13.66	92.54	1.27
14	250	100	32	0	2	21.13	0.12	44.50	1.57
15	175	175	16	0.4	1.25	32.52	2.65	91.05	1.63
16	250	100	32	0.8	0.5	58.26	13.40	94.45	1.28
17	175	175	24	0	1.25	29.88	1.57	68.95	1.78
18	100	175	24	0.4	1.25	19.98	0.09	58.70	1.45
19	175	175	24	0.4	0.5	53.60	9.70	93.83	1.13
20	250	100	16	0	0.5	50.75	8.42	95.88	1.25
21	250	250	32	0.8	2	23.24	0.50	49.07	1.62
22	175	250	24	0.4	1.25	32.91	2.67	72.48	1.71
23	175	175	32	0.4	1.25	27.64	1.02	58.93	1.78
24	175	100	24	0.4	1.25	25.14	0.43	58.08	1.71
25	250	100	16	0.8	0.5	53.38	10.52	96.52	1.22
26	100	250	32	0.8	2	14.61	0.00	9.36	1.24
27	250	250	16	0	0.5	61.62	13.04	95.29	1.03
28	250	250	16	0.8	2	29.69	1.60	78.62	1.72
29	175	175	24	0.4	1.25	30.12	1.68	67.97	1.81
30	100	250	32	0	2	14.84	0.00	23.15	1.26
31	250	175	24	0.4	1.25	34.47	2.92	63.25	1.93
32	250	250	32	0	2	28.56	1.99	50.49	1.67
33	100	100	32	0.8	2	14.14	0.00	21.61	1.29
34	250	100	16	0	2	22.57	0.18	68.27	1.69
35	100	100	16	0	0.5	29.56	0.57	97.10	1.20
36	100	100	16	0.8	2	14.53	0.00	56.18	1.30
37	100	100	32	0.8	0.5	32.79	0.93	96.27	1.23
38	100	250	32	0	0.5	44.65	5.12	91.71	1.15
39	100	250	32	0.8	0.5	43.85	5.36	95.03	1.16
40	100	100	16	0.8	0.5	28.94	0.40	98.05	1.15
41	100	100	32	0	0.5	34.26	1.73	92.50	1.34
42	100	250	16	0	2	16.55	0.00	48.23	1.32
43	250	250	16	0.8	0.5	58.92	12.11	94.03	1.04

Independent parameters include width (*w*), thickness (*t*), number of struts (NS), link amplitude (LA), and strut amplitude (SA). The response values are mean intramural stress, proportion of the artery with Von Mises stress exceeding 100 kPa, stent-artery apposition, and vessel lumen pinching

**Table 5:** The central composite matrix of independent variables and their response values for the second DOE

Exp. No.	Experimental layout			Response values			
		
	*w*, μm	t, μm	SA, mm	Mean stress, kPa	High-stress area, %	Apposition, %	Pinching

1	150	150	0.7	40.08	5.01	94.08	1.24
2	50	50	0.7	17.24	0.00	79.36	1.24
3	150	50	0.7	22.89	0.16	91.81	1.23
4	100	50	0.5	22.30	0.01	93.24	1.11
5	100	150	0.5	34.09	2.40	94.80	1.12
6	100	100	0.3	35.23	1.80	96.14	1.09
7	150	50	0.3	36.55	2.32	92.47	1.36
8	100	100	0.7	23.03	0.20	94.85	1.20
9	150	150	0.3	52.11	8.70	97.18	1.07
10	150	100	0.5	39.85	4.48	94.22	1.21
11	50	50	0.3	20.48	0.00	92.31	1.39
12	100	100	0.5	29.04	0.60	96.41	1.14
13	50	150	0.7	17.25	0.00	93.44	1.26
14	50	100	0.5	19.46	0.00	93.96	1.24
15	50	150	0.3	26.97	0.05	100.00	1.09

Experimental layout include width (*w*), thickness (*t*), and strut amplitude (SA). The response values are mean intramural stress, proportion of the artery with Von Mises stress exceeding 100 kPa, stent-artery apposition, and vessel lumen pinching.

**Table 6:** Optimized stent parameters according to the first and second DOE, along with their response values

Optimized designs	Experimental layout					Response values			
		
	*w*, μm	t, μm	NS	LA, mm	SA, mm	Mean stress, kPa	High-stress area, %	Apposition, %	Pinching

DOE 1	100	100	16	0.8	0.5	28.9	0.4	98.1	1.15
DOE 2	70	140	16*	0.4*	0.5	25.2	0.1	96.4	1.13

Parameters marked with * were not optimized in DOE2 but were set based on DOE1.

## Data Availability

The datasets used and/or analyzed during the current study are available from the corresponding author on reasonable request.

## Appendix A

See Figs. 7, 8, 9 and 10

## References

[1] Dieter RS, Chu WW, Pacanowski JP, McBride PE, and Tanke TE The significance of lower extremity peripheral arterial disease. Clin Cardiol. 25(1):3–10, 2002. 10.1002/CLC.4950250103. 11808837 PMC6654368

[2] Salisbury AC, and Cohen DJ Economic analysis in peripheral artery disease. Endovasc Today. 15(10):53–57, 2016.

[3] Mahoney EM, Wang K, Keo HH, Duval S, Smolderen KG, Cohen DJ, Steg G, Bhatt DL, Hirsch AT, Investigators* on behalf of the R of A for CH (REACH) R. Vascular Hospitalization Rates and Costs in Patients With Peripheral Artery Disease in the United States. Circ Cardiovasc Qual Outcomes. 2010;3(6):642–651. 10.1161/CIRCOUTCOMES.109.930735 20940249

[4] Conte MS, Bandyk DF, Clowes AW, Moneta GL, Seely L, Lorenz TJ, Namini H, Hamdan AD, Roddy SP, Belkin M, Berceli SA, DeMasi RJ, Samson RH, Berman SS. Results of PREVENT III: A multicenter, randomized trial of edifoligide for the prevention of vein graft failure in lower extremity bypass surgery. J Vasc Surg. 2006;43(4). 10.1016/j.jvs.2005.12.058 16616230

[5] Schillinger M, Sabeti S, Loewe C, Dick P, Amighi J, Mlekusch W, Schlager O, Cejna M, Lammer J, Minar E. Balloon Angioplasty versus Implantation of Nitinol Stents in the Superficial Femoral Artery. 2009;354(18):1879–1888. 10.1056/NEJMOA051303 16672699

[6] Schillinger M, Sabeti S, Dick P, Amighi J, Mlekusch W, Schlager O, Loewe C, Cejna M, Lammer J, and Minar E Sustained benefit at 2 years of primary femoropopliteal stenting compared with balloon angioplasty with optional stenting. Circulation. 115(21):2745–2749, 2007. 17502568 10.1161/CIRCULATIONAHA.107.688341

[7] Poulson W, Kamenskiy A, Seas A, Deegan P, Lomneth C, and MacTaggart J Limb flexion-induced axial compression and bending in human femoropopliteal artery segments. J Vasc Surg. 67(2):607–613, 2018. 28526560 10.1016/j.jvs.2017.01.071 PMC5690897

[8] Laird JR, Yeo KK. The treatment of femoropopliteal in-stent restenosis: back to the future. American College of Cardiology Foundation Washington, DC. Preprint posted online 2012. 10.1016/j.jacc.2011.09.037 22192664

[9] Farber A, Menard MT, Conte MS, Kaufman JA, Powell RJ, Choudhry NK, Hamza TH, Assmann SF, Creager MA, Cziraky MJ, Dake MD, Jaff MR, Reid D, Siami FS, Sopko G, White CJ, van Over M, Strong MB, Villarreal MF, McKean M, Azene E, Azarbal A, Barleben A, Chew DK, Clavijo LC, Douville Y, Findeiss L, Garg N, Gasper W, Giles KA, Goodney PP, Hawkins BM, Herman CR, Kalish JA, Koopmann MC, Laskowski IA, Mena-Hurtado C, Motaganahalli R, Rowe VL, Schanzer A, Schneider PA, Siracuse JJ, Venermo M, and Rosenfield K Surgery or Endovascular Therapy for Chronic Limb-Threatening Ischemia. N Engl J Med. 387(25):2305–2316, 2022. 10.1056/nejmoa2207899. 36342173

[10] Haraguchi T, Kuramitsu S, Tsujimoto M, Kashima Y, Sato K, and Fujita T Outcomes of non-flow-limiting spiral dissection after drug-coated balloon angioplasty for de novo femoropopliteal lesions. Catheter Cardiovasc Interv. 103(1):97–105, 2024. 10.1002/ccd.30911. 37975201

[11] Bosiers M, Scheinert D, Hendriks JMH, Wissgott C, Peeters P, Zeller T, Brodmann M, Staffa R, Deloose K, de Vries JPPM, van den Heuvel DAF, Krankenberg H, Kubicek L, Dorr A, Gissler HM, Schmidt A, Braunlich S, Ulrich M, Schuster J, Scheinert S, Bausback Y, Piorkowski M, Werner M, Verbist J, Maene L, Beelen R, Schwarzwälder U, Rastan A, Beschorner U, Sixt S, Pilger E, Eller P, Ricke J, Pech M, Ludtke C, Vojtisek B, and Vlachovsky R Results from the Tack Optimized Balloon Angioplasty (TOBA) study demonstrate the benefits of minimal metal implants for dissection repair after angioplasty. J Vasc Surg. 64(1):109–116, 2016. 10.1016/j.jvs.2016.02.043. 27139789

[12] Laird JR, Katzen BT, Scheinert D, Lammer J, Carpenter J, Buchbinder M, Dave R, Ansel G, Lansky A, Cristea E, Collins TJ, Goldstein J, and Jaff MR Nitinol stent implantation versus balloon angioplasty for lesions in the superficial femoral artery and proximal popliteal artery: twelve-month results from the RESILIENT randomized trial. Circ Cardiovasc Interv. 3(3):267–276, 2010. 10.1161/CIRCINTERVENTIONS.109.903468. 20484101

[13] Siracuse JJ, Giles KA, Pomposelli FB, Hamdan AD, Wyers MC, Chaikof EL, Nedeau AE, and Schermerhorn ML Results for primary bypass versus primary angioplasty/stent for intermittent claudication due to superficial femoral artery occlusive disease. J Vasc Surg. 55(4):1001–1007, 2012. 10.1016/j.jvs.2011.10.128. 22301210 PMC3319263

[14] Stavroulakis K, Torsello G, Manal A, Schwindt A, Hericks C, Stachmann A, Schönefeld E, and Bisdas T Results of primary stent therapy for femoropopliteal peripheral arterial disease at 7 years. J Vasc Surg. 64(6):1696–1702, 2016. 10.1016/j.jvs.2016.05.073. 27575816

[15] Laird JR, Yeo KK. The Treatment of Femoropopliteal In-Stent Restenosis Back to the Future*. Published online 2012. 10.1016/j.jacc.2011.09.037 22192664

[16] Qato K, Conway AM, Mondry L, Giangola G, and Carroccio A Management of isolated femoropopliteal in-stent restenosis. J Vasc Surg. 68(3):807–810, 2018. 10.1016/j.jvs.2018.01.030. 30144908

[17] Schillinger M, Sabeti S, Dick P, Amighi J, Mlekusch W, Schlager O, Loewe C, Cejna M, Lammer J, and Minar E Sustained benefit at 2 years of primary femoropopliteal stenting compared with balloon angioplasty with optional stenting. Circulation. 115(21):2745–2749, 2007. 10.1161/CIRCULATIONAHA.107.688341. 17502568

[18] Gouëffic Y, Torsello G, Zeller T, Esposito G, Vermassen F, Hausegger KA, Tepe G, Thieme M, Gschwandtner M, Kahlberg A, Schindewolf M, Sapoval M, Diaz-Cartelle J, Stavroulakis K, on behalf of the EMINENT Investigators. Efficacy of a Drug-Eluting Stent Versus Bare Metal Stents for Symptomatic Femoropopliteal Peripheral Artery Disease: Primary Results of the EMINENT Randomized Trial. Circulation. 2022;146(21):1564–1576. 10.1161/CIRCULATIONAHA.122.059606 36254728

[19] Gouëffic Y, Brodmann M, Deloose K, Dubosq-Lebaz M, and Nordanstig J Drug-eluting devices for lower limb peripheral arterial disease. EuroIntervention. 20(18):e1136–e1153, 2024. 10.4244/EIJ-D-23-01080. 39279515 PMC11423351

[20] Bisdas T, Beropoulis E, Argyriou A, Torsello G, and Stavroulakis K 1-Year All-Comers Analysis of the Eluvia Drug-Eluting Stent for Long Femoropopliteal Lesions After Suboptimal Angioplasty. JACC Cardiovasc Interv. 11(10):957–966, 2018. 10.1016/j.jcin.2018.03.046. 29798772

[21] Cortese B Paclitaxel-Eluting Stents and Aneurysm Formation, A Worrisome Association∗. JACC Cardiovasc Interv. 11(10):967–968, 2018. 10.1016/j.jcin.2018.04.024. 29798773

[22] Nordanstig J, James S, Andersson M, Andersson M, Delle M, Engström J, Fransson T, Gillgren P, Hilbertson A, Hörer TM, Jacobsson E, Kragsterman B, Lindbäck J, Lindgren H, Ludwigs K, Mellander S, Nelzén O, Olin R, Sigvant B, Skoog P, Starck J, Tegler G, Thorbjørnsen K, Truedson M, Wahlgren CM, Wallinder J, Öjersjö A, and Falkenberg M Paclitaxel-coated versus uncoated devices for infrainguinal endovascular revascularisation in patients with intermittent claudication (SWEDEPAD 2): a multicentre, participant-masked, registry-based, randomised controlled trial. The Lancet. 406(10508):1115–1127, 2025. 10.1016/S0140-6736(25)01584-3. 40902614

[23] Falkenberg M, James S, Andersson M, Andersson M, Delle M, Engström J, Fransson T, Gillgren P, Hilbertson A, Hörer TM, Jacobsson E, Kragsterman B, Lindbäck J, Lindgren H, Ludwigs K, Mellander S, Nelzén O, Olin R, Sigvant B, Skoog P, Starck J, Tegler G, Thorbjørnsen K, Truedson M, Wahlgren CM, Wallinder J, Öjersjö A, and Nordanstig J Paclitaxel-coated versus uncoated devices for infrainguinal endovascular revascularisation in chronic limb-threatening ischaemia (SWEDEPAD 1): a multicentre, participant-masked, registry-based, randomised controlled trial. The Lancet. 406(10508):1103–1114, 2025. 10.1016/S0140-6736(25)01585-5. 40902617

[24] Papafaklis MI, Chatzizisis YS, Naka KK, Giannoglou GD, and Michalis LK Drug-eluting stent restenosis: Effect of drug type, release kinetics, hemodynamics and coating strategy. Pharmacol Ther. 134(1):43–53, 2012. 10.1016/j.pharmthera.2011.12.006. 22212618

[25] Maleckis K, Anttila E, Aylward P, Poulson W, Desyatova A, MacTaggart J, and Kamenskiy A Nitinol stents in the femoropopliteal artery: a mechanical perspective on material, design, and performance. Ann Biomed Eng. 46(5):684–704, 2018. 29470746 10.1007/s10439-018-1990-1 PMC5975366

[26] Vogel J, Berg BT, Dawson J, Kamenskiy A. Flexible Implantable Device Shape History. In: Baxter W, Lahm R, eds. Measuring the Physiologic Use Conditions of Medical Devices. Springer International Publishing; 2024:125–160. 10.1007/978-3-031-62764-4_7

[27] MacTaggart JN, Phillips NY, Lomneth CS, Pipinos II, Bowen R, Baxter BT, Johanning J, Longo GM, Desyatova AS, and Moulton MJ Three-dimensional bending, torsion and axial compression of the femoropopliteal artery during limb flexion. J Biomech. 47(10):2249–2256, 2014. 24856888 10.1016/j.jbiomech.2014.04.053

[28] Desyatova A, Poulson W, Deegan P, Lomneth C, Seas A, Maleckis K, MacTaggart J, Kamenskiy A. Limb flexion-induced twist and associated intramural stresses in the human femoropopliteal artery. J R Soc Interface. 2017;14(128). 10.1098/rsif.2017.0025 PMC5378143 28330991

[29] Desyatova A, Poulson W, MacTaggart J, Maleckis K, Kamenskiy A. Cross-sectional pinching in human femoropopliteal arteries due to limb flexion, and stent design optimization for maximum cross-sectional opening and minimum intramural stresses. J R Soc Interface. 2018;15(145). 10.1098/rsif.2018.0475 PMC6127163 30135264

[30] Cheng CP, Wilson NM, Hallett RL, Herfkens RJ, and Taylor CA In Vivo MR Angiographic Quantification of Axial and Twisting Deformations of the Superficial Femoral Artery Resulting from Maximum Hip and Knee Flexion. J Vasc Interv Radiol. 17(6):979–987, 2006. 10.1097/01.RVI.0000220367.62137.E8. 16778231

[31] Cheng CP, Choi G, Herfkens RJ, and Taylor CA The Effect of Aging on Deformations of the Superficial Femoral Artery Resulting from Hip and Knee Flexion: Potential Clinical Implications. J Vasc Interv Radiol. 21(2):195–202, 2010. 10.1016/J.JVIR.2009.08.027. 20022767 PMC2818320

[32] Klein AJ, James Chen S, Messenger JC, Hansgen AR, Plomondon ME, Carroll JD, and Casserly IP Quantitative assessment of the conformational change in the femoropopliteal artery with leg movement. Catheter Cardiovasc Interv. 74(5):787–798, 2009. 19521998 10.1002/ccd.22124

[33] Ansari F, Pack LK, Brooks SS, and Morrison TM Design considerations for studies of the biomechanical environment of the femoropopliteal arteries. J Vasc Surg. 58(3):804–813, 2013. 10.1016/j.jvs.2013.03.052. 23870198

[34] Maleckis K, Deegan P, Poulson W, Sievers C, Desyatova A, MacTaggart J, and Kamenskiy A Comparison of femoropopliteal artery stents under axial and radial compression, axial tension, bending, and torsion deformations. J Mech Behav Biomed Mater. 75:160–168, 2017. 28734257 10.1016/j.jmbbm.2017.07.017 PMC5623954

[35] MacTaggart J, Poulson W, Seas A, Deegan P, Lomneth C, Desyatova A, Maleckis K, and Kamenskiy A Stent Design Affects Femoropopliteal Artery Deformation. Ann Surg. 270(1):180–187, 2019. 10.1097/SLA.0000000000002747. 29578912 PMC6151294

[36] Keiser C, Maleckis K, Struczewska P, Jadidi M, MacTaggart J, Kamenskiy A. A method of assessing peripheral stent abrasiveness under cyclic deformations experienced during limb movement. Acta Biomater. 2022;Online ahe(Sep 24;S1742–7061(22)00613–4). 10.1016/j.actbio.2022.09.044 PMC9810438 36162765

[37] Schillinger M, Minar E. Past, Present and Future of Femoropopliteal Stenting. J Endovasc Ther. 2009;16(1_suppl):147–152. 10.1583/1545-1550-16.16.I-147 19317587

[38] Adlakha S, Sheikh M, Wu J, Burket MW, Pandya U, Colyer W, Eltahawy E, and Cooper CJ Stent fracture in the coronary and peripheral arteries. J Intervent Cardiol. 23(4):411–419, 2010. 20806458 10.1111/j.1540-8183.2010.00567.x

[39] Aw C, Ma R, and Mm C Mechanisms of stenosis after arterial injury. Lab Investig J Tech Methods Pathol. 49(2):208–215, 1983. 6876748

[40] Wensing PJW, Meiss L, Mali WPTM, and Hillen B Early atherosclerotic lesions spiraling through the femoral artery. Arterioscler Thromb Vasc Biol. 18(10):1554–1558, 1998. 9763526 10.1161/01.atv.18.10.1554

[41] Dottori S, Flamini V, and Vairo G Mechanical behavior of peripheral stents and stent-vessel interaction: A computational study. Int J Comput Methods Eng Sci Mech. 17(3):196–210, 2016. 10.1080/15502287.2016.1188530.

[42] Stojkovic S, Jurisic M, Kopp CW, Koppensteiner R, Huber K, Wojta J, and Gremmel T Circulating microRNAs identify patients at increased risk of in-stent restenosis after peripheral angioplasty with stent implantation. Atherosclerosis. 269:197–203, 2018. 10.1016/j.atherosclerosis.2018.01.020. 29366993

[43] Fischman DL, Leon MB, Baim DS, Schatz RA, Savage MP, Penn I, Detre K, Veltri L, Ricci D, and Nobuyoshi M A randomized comparison of coronary-stent placement and balloon angioplasty in the treatment of coronary artery disease. N Engl J Med. 331(8):496–501, 1994. 8041414 10.1056/NEJM199408253310802

[44] Cheneau E, Leborgne L, Mintz GS, Kotani J ichi, Pichard AD, Satler LF, Canos D, Castagna M, Weissman NJ, Waksman R. Predictors of Subacute Stent Thrombosis. Circulation. 2003;108(1). 10.1161/01.cir.0000078636.71728.40 12821553

[45] Dierk S, Susanne S, Jacqueline S, Christopher P, Sven B, Matthias U, Giancarlo B, and Andrej S Prevalence and clinical impact of stent fractures after femoropopliteal stenting. J Am Coll Cardiol. 45(2):312–315, 2005. 10.1016/j.jacc.2004.11.026. 15653033

[46] Atsushi T, Yoshimitsu S, Osamu I, Takayuki I, Keisuke H, Kenji S, Hiroyoshi Y, Shinsuke N, and Masakiyo N Classification and Clinical Impact of Restenosis After Femoropopliteal Stenting. J Am Coll Cardiol. 59(1):16–23, 2012. 10.1016/j.jacc.2011.09.036. 22192663

[47] Soga Y, Iida O, Hirano K, Yokoi H, Nanto S, and Nobuyoshi M Mid-term clinical outcome and predictors of vessel patency after femoropopliteal stenting with self-expandable nitinol stent. J Vasc Surg. 52(3):608–615, 2010. 10.1016/J.JVS.2010.03.050. 20573476

[48] Early M, Lally C, Prendergast PJ, and Kelly DJ Stresses in peripheral arteries following stent placement: a finite element analysis. Comput Methods Biomech Biomed Engin. 12(1):25–33, 2009. 18821189 10.1080/10255840903065043

[49] Gökgöl C, Diehm N, Nezami FR, Büchler P. Nitinol Stent Oversizing in Patients Undergoing Popliteal Artery Revascularization: A Finite Element Study. Ann Biomed Eng. 2015;43(12). 10.1007/s10439-015-1358-8 26101031

[50] Kleinstreuer C, Li Z, Basciano CA, Seelecke S, Farber MA. Computational mechanics of Nitinol stent grafts. J Biomech. 2008;41(11). 10.1016/j.jbiomech.2008.05.032 18644312

[51] He R, Zhao L, Silberschmidt VV., Willcock H, Vogt F. Pioneering personalised design of femoropopliteal nitinol stents. Mater Sci Eng C. 2021;130. 10.1016/j.msec.2021.112462 34702537

[52] Anttila E, Balzani D, Desyatova A, Deegan P, MacTaggart J, and Kamenskiy A Mechanical damage characterization in human femoropopliteal arteries of different ages. Acta Biomater. 90:225–240, 2019. 10.1016/J.ACTBIO.2019.03.053. 30928732 PMC6532398

[53] Swift H, Bordoni B. Anatomy, Bony Pelvis and Lower Limb: Femoral Artery. In: StatPearls. StatPearls Publishing; 2025. Accessed January 9, 2025. http://www.ncbi.nlm.nih.gov/books/NBK538262/

[54] Jadidi M, Desyatova A, MacTaggart J, and Kamenskiy A Mechanical stresses associated with flattening of human femoropopliteal artery specimens during planar biaxial testing and their effects on the calculated physiologic stress-stretch state. Biomech Model Mechanobiol. 18(6):1591–1605, 2019. 10.1007/s10237-019-01162-0. 31069592 PMC7191998

[55] Shahbad R, Pipinos M, Jadidi M, Desyatova A, Gamache J, MacTaggart J, Kamenskiy A. Structural and Mechanical Properties of Human Superficial Femoral and Popliteal Arteries. Ann Biomed Eng. Published online February 6, 2024. 10.1007/s10439-023-03435-3 PMC11455778 38321357

[56] Desyatova A, MacTaggart J, Poulson W, Deegan P, Lomneth C, Sandip A, Kamenskiy A. The choice of a constitutive formulation for modeling limb flexion-induced deformations and stresses in the human femoropopliteal arteries of different ages. Biomech Model Mechanobiol. 2017;16(3). 10.1007/s10237-016-0852-8 PMC5423836 27868162

[57] Desyatova A, Mactaggart J, Romarowski R, Poulson W, Conti M, Kamenskiy A. Effect of aging on mechanical stresses, deformations, and hemodynamics in human femoropopliteal artery due to limb flexion. Biomech Model Mechanobiol. 2018;17(1). 10.1007/s10237-017-0953-z PMC5809209 28815378

[58] Watt JK Origin of femoro-popliteal occlusions. Br Med J. 2(5476):1455, 1965. 5849435 10.1136/bmj.2.5476.1455 PMC1847035

[59] Kamenskiy A, Seas A, Bowen G, Deegan P, Desyatova A, Bohlim N, Poulson W, and Mactaggart J In situ longitudinal pre-stretch in the human femoropopliteal artery. Acta Biomater. 32:231–237, 2016. 10.1016/j.actbio.2016.01.002. 26766633 PMC4889118

[60] Holzapfel GA, Gasser TC, Ogden RW. A new constitutive framework for arterial wall mechanics and a comparative study of material models. J Elast. 2000;61(1–3). 10.1023/A:1010835316564

[61] Kamenskiy A, Seas A, Deegan P, Poulson W, Anttila E, Sim S, Desyatova A, MacTaggart J. Constitutive description of human femoropopliteal artery aging. Biomech Model Mechanobiol. 2017;16(2). 10.1007/s10237-016-0845-7 PMC5352506 27771811

[62] Lally C, Dolan F, and Prendergast PJ Cardiovascular stent design and vessel stresses: a finite element analysis. J Biomech. 38(8):1574–1581, 2005. 10.1016/j.jbiomech.2004.07.022. 15958213

[63] Anderson MJ, Whitcomb PJ. RSM Simplified: Optimizing Processes Using Response Surface Methods for Design of Experiments. Productivity press; 2016.

[64] Harrington EC The desirability function. Ind Qual Control. 21(10):494–498, 1965.

[65] Derringer G, and Suich R Simultaneous optimization of several response variables. J Qual Technol. 12(4):214–219, 1980.

[66] Higashiura W, Kubota Y, Sakaguchi S, Kurumatani N, Nakamae M, Nishimine K, and Kichikawa K Prevalence, factors, and clinical impact of self-expanding stent fractures following iliac artery stenting. J Vasc Surg. 49(3):645–652, 2009. 10.1016/J.JVS.2008.10.019. 19268770

[67] Azaouzi M, Makradi A, and Belouettar S Deployment of a self-expanding stent inside an artery: a finite element analysis. Mater Des. 41:410–420, 2012.

[68] Wressnegger A, Kaider A, and Funovics MA Self-expanding nitinol stents of high versus low chronic outward force in de novo femoropopliteal occlusive arterial lesions (BIOFLEX-COF trial): study protocol for a randomized controlled trial. Trials. 18(1):594, 2017. 10.1186/s13063-017-2338-0. 29237489 PMC5729260

[69] Li H, Rha SW, Choi BG, Choi SY, Moon SK, Jang WY, Kim W, Ahn JH, Park SH, Choi WG, Yang RF, Bai WW, Choi CU, Ryu Y gi, Baek MJ, Oh DJ. Impact of chronic outward force on arterial responses of proximal and distal of long superficial femoral artery stent. BMC Cardiovasc Disord. 2021;21:323. 10.1186/s12872-021-02141-z 34193057 PMC8246708

[70] LaDisa JF Jr., Olson LE, Guler I, Hettrick DA, Audi SH, Kersten JR, Warltier DC, and Pagel PS Stent design properties and deployment ratio influence indexes of wall shear stress: a three-dimensional computational fluid dynamics investigation within a normal artery. J Appl Physiol. 97(1):424–430, 2004. 14766776 10.1152/japplphysiol.01329.2003

[71] Shen X, Yi H, Ni Z. Effects of Stent Design Parameters on Radial Force of Stent. In: 2008 2nd International Conference on Bioinformatics and Biomedical Engineering. 2008:1712–1716. 10.1109/ICBBE.2008.756

[72] Bedoya J, Meyer CA, Timmins LH, Moreno MR, and Moore JE Jr. Effects of Stent Design Parameters on Normal Artery Wall Mechanics. J Biomech Eng. 128(5):757–765, 2006. 10.1115/1.2246236. 16995763

[73] Noad RL, Hanratty C, and Walsh SJ Clinical Impact of Stent Design. Interv Cardiol Rev. 9(2):89–93, 2014. 10.15420/icr.2011.9.2.89. PMC5808536 29588784

[74] Kamenskiy A, de Oliveira B, Heinis F, Renavikar P, Eberth J, and MacTaggart J Large Animal Model of Controlled Peripheral Artery Calcification. Acta Biomater. 199:301–314, 2025. 40328616 10.1016/j.actbio.2025.04.055 PMC12513339

